# HMGB1 is a Central Driver of Dynamic Pro-inflammatory Networks in Pediatric Acute Liver Failure induced by Acetaminophen

**DOI:** 10.1038/s41598-019-42564-5

**Published:** 2019-04-12

**Authors:** Ruben Zamora, Derek Barclay, Jinling Yin, Estella M. Alonso, Mike A. Leonis, Qi Mi, Timothy R. Billiar, Richard L. Simmons, Robert H. Squires, Yoram Vodovotz

**Affiliations:** 1 Department of Surgery, University of Pittsburgh, Pittsburgh, PA 15213 USA; 2 Center for Inflammation and Regenerative Modeling, McGowan Institute for Regenerative Medicine, Pittsburgh, PA 15219 USA; 3 Ann & Robert H. Lurie Children’s Hospital of Chicago, Chicago, IL 60611 USA; 4 Department of Pediatrics, University of Alabama at Birmingham, Birmingham, AL 35233 USA; 5 Department of Pediatrics, University of Pittsburgh, Pittsburgh, PA 15213 USA

## Abstract

Acetaminophen (APAP) overdose (APAPo) is predominant in the NIH Pediatric Acute Liver Failure (PALF) Study. We assayed multiple inflammatory mediators in serial serum samples from 13 PALF survivors with APAPo + N-acetylcysteine (NAC, the frontline therapy for APAPo), 8 non-APAPo + NAC, 40 non-APAPo non-NAC, and 12 non-survivors. High Mobility Group Box 1 (HMGB1) was a dominant mediator in dynamic inflammation networks in all sub-groups, associated with a threshold network complexity event at d1–2 following enrollment that was exceeded in non-survivors vs. survivors. We thus hypothesized that differential HMGB1 network connectivity after day 2 is related to the putative threshold event in non-survivors. DyNA showed that HMGB1 is most connected in non-survivors on day 2–3, while no connections were observed in APAPo + NAC and non-APAPo + NAC survivors. Inflammatory dynamic networks, and in particular HMGB1 connectivity, were associated with the use of NAC in the context of APAPo. To recapitulate hepatocyte (HC) damage *in vitro*, primary C57BL/6 HC and HC-specific HMGB1-null HC were treated with APAP + NAC. Network phenotypes of survivors were recapitulated in C57BL/6 mouse HC and were greatly altered in HMGB1-null HC. HC HMGB1 may thus coordinate a pro-inflammatory program in PALF non-survivors (which is antagonized by NAC), while driving an anti-inflammatory/repair program in survivors.

## Introduction

Pediatric Acute Liver Failure (PALF) is a life-threatening clinical syndrome with poor outcomes and where one-half of patients die or receive liver transplantation (LTx)^1^. The etiology of PALF and its different clinical manifestations are very complex and still poorly understood. The precise onset of disease is rarely identified, with an exception being acute ingestions (e.g., mushrooms, acetaminophen)^1^. PALF has a dynamic and multifactorial clinical trajectory, and the outcomes vary among children with seemingly similar etiology, disease severity, and treatment; thus, additional factors are likely involved to explain these variations. Such factors likely include a complex interaction among the inflammatory milieu, end-organ damage, immune activation, potential for liver regeneration, and interventions.

Acetaminophen (APAP) toxicity, commonly due to APAP overdose (APAPo) is the most common identifiable cause of Acute Liver Failure (ALF) in both children^2^ and adults^3^. Liver injury due to APAP toxicity occurs when inherent mechanisms to detoxify APAP – including conjugation with glutathione – are overwhelmed, resulting in the formation of reactive oxygen species that form destructive adducts with vital intracellular proteins. N-acetyl cysteine (NAC) serves to replete glutathione stores and is the established treatment for acute APAP toxicity. However, while NAC is a clinically accepted treatment of acute liver injury due to APAP toxicity, a potential mechanism to support its use in non-APAP ALF has not been established^4^.

High-mobility group box-1 (HMGB1) is an evolutionarily conserved, chromatin-binding protein expressed in virtually all types of cells^5^. Cells that are undergoing necrosis passively release HMGB1 into the extracellular space, where HMGB1 works as a damage-associated molecular pattern (DAMP) molecule. HMGB1 is also a potential mediator of APAP-induced hepatotoxicity, and anti-HMGB1 antibodies and knocking out HMGB1 in the liver can reduce hepatic inflammation and liver injury in mouse models of APAP toxicity^6^.

We hypothesized that differential HMGB1 network connectivity is related to a putative threshold event that differentiate PALF patients. In addition, we also hypothesized that key inflammatory mediators and networks suggested from our computational analyses may differentiate patient etiologies and suggest novel therapeutic opportunities for PALF. To address these hypotheses, a panel of relevant serum mediators, including HMGB1, were measured in PALF participants. We focused on HMGB1 as a proximal mediator of interest because of its known role as a driver of inflammation in models of APAP-induced liver injury^7^. As an extension of our previous work^8,9^, Translational Systems Biology modeling methodology developed for biologically complex and dynamic conditions^10,11^ was applied to correlate biomarkers with clinical outcomes as well as identify potential therapeutic targets in PALF. Recapitulation of these findings using an *in vitro* model of isolated HC exposed to APAP ± NAC was used to create a bedside-to-bench translational platform for further investigation.

## Results

### Time-courses of inflammatory mediators in PALF survivors vs. non-survivors

**Figure 1:**
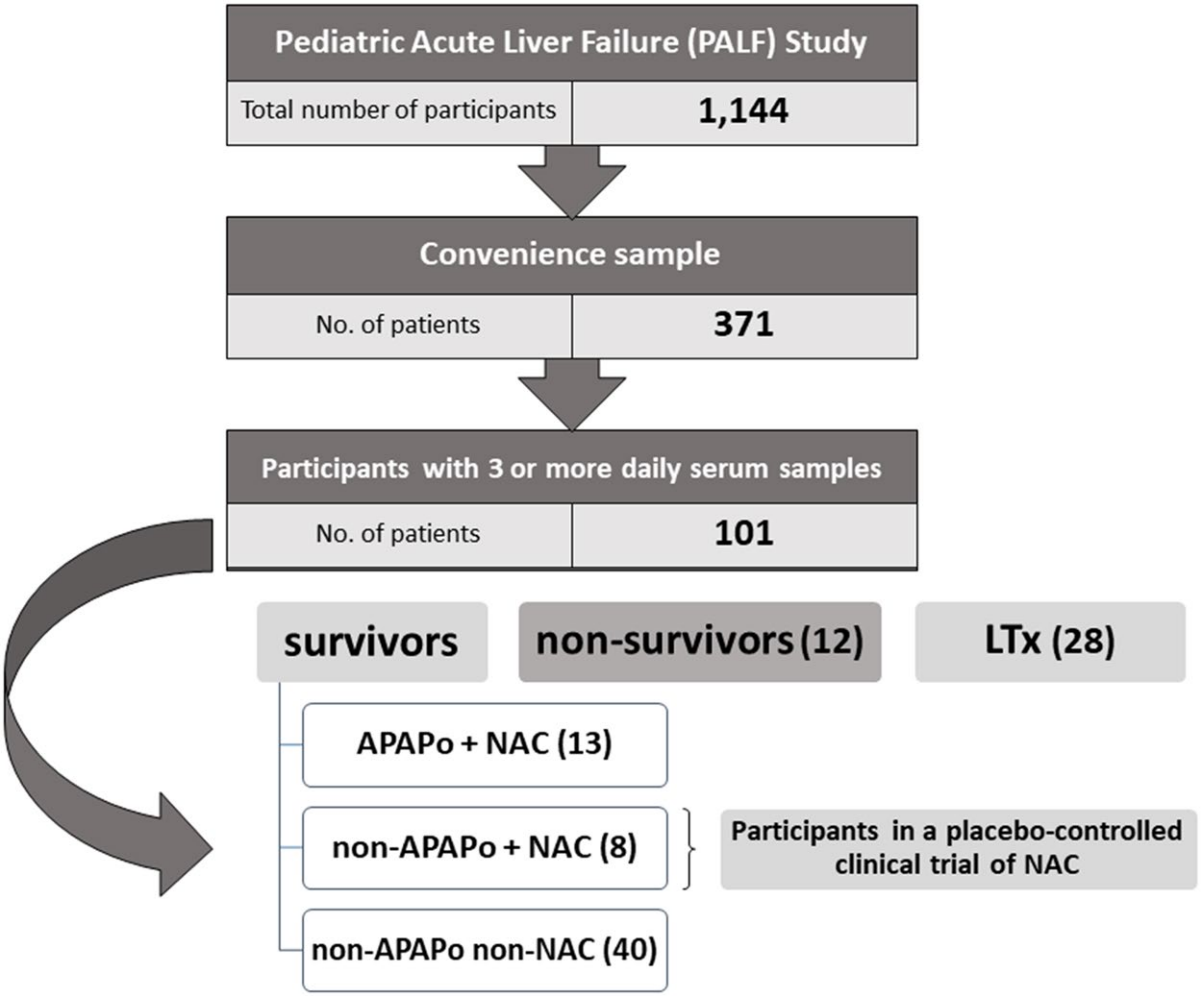
Flow chart of recruitment and PALF study participation. From a large cohort of 1,144 participants in the PALF study, an initial Convenience Sample was identified consisting of 371 participants with serum samples collected per protocol for analysis of immune and inflammatory markers. Participants were further selected (n = 101) if they had at least three daily samples with at least 100 µl of serum available. Following exclusion of 28 participants who received a LTx, selection criteria were met by 73 participants and clinical outcomes at 21 days following enrollment were assigned as indicated.

To assess the response and time-dependent changes in inflammatory mediators across the PALF non-survivors and three survivor sub-groups (APAPo + NAC, non-APAPo + NAC, and non-APAPo non-NAC) (Fig. 1), we assayed a number of mediators that represent most of the major inflammatory and immune pathways. All inflammatory mediators varied over time in a complex fashion. Furthermore, all mediators except **I**L-10, MIP-1β, and HMGB1, differed significantly over time as a function of patient sub-group (Suppl. Fig. 1). This analysis suggested that the standard statistical analysis of mediators, singly or as a group, may be of limited use for characterizing and predicting patient outcomes, because of potentially more complex, non-linear, and non-intuitive interactions among inflammatory mediators. Thus, we next utilized computational analyses to study the PALF patient sub-groups.

### Dynamic Bayesian Network (DBN) analysis suggests a central role for HMGB1 in PALF

**Figure 2:**
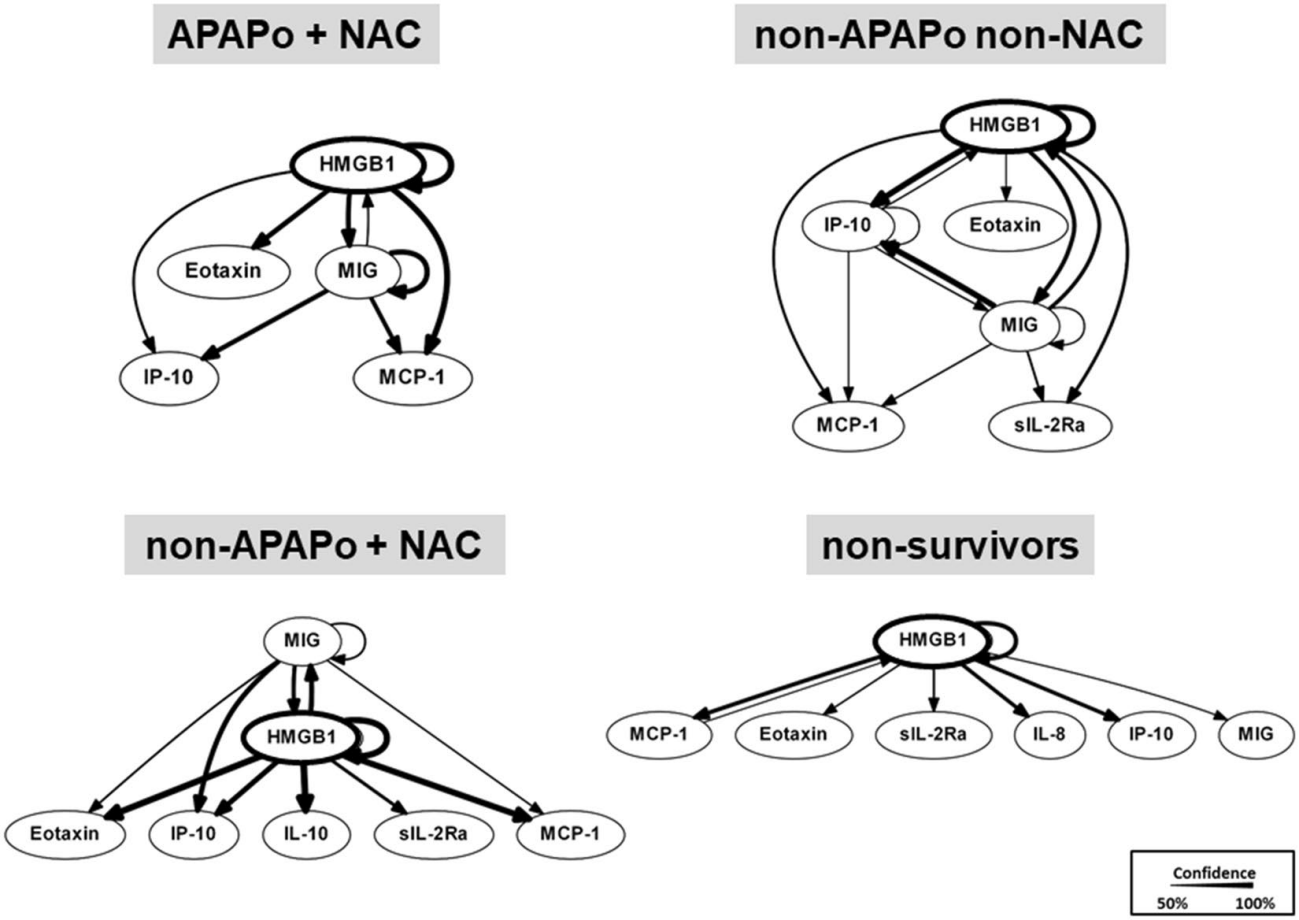
Dynamic Bayesian Network (DBN) analysis of circulating inflammatory mediators in PALF patients. Circulating inflammatory mediators in serum samples from PALF spontaneous survivors were segregated into three sub-groups (APAPo + NAC [n = 13 patients], non-APAPo + NAC [n = 8 patients], and non-APAPo non-NAC [n = 40 patients]) and non-survivors (n = 12 patients). DBN inference was performed as described in *Materials and Methods*. Inflammatory mediators are shown as nodes, and the arrows connecting them suggest an influence of one mediator on the one(s) to which it is connected. The arrows do not distinguish positive from negative influences of one mediator on another. Semi-circular arrows suggest either positive or negative feedback of a given mediator on itself. The thickness of each edge denotes the relative algorithmic confidence in a given interaction between nodes.

We next hypothesized that computational analysis would point us to the central inflammatory mediators and pathways associated with specific PALF patient sub-groups. Similar to our previous studies in PALF^8,9^, we utilized DBN inference to determine such network structures that could be discerned from the time courses of circulating inflammatory mediators in PALF participants. Central nodes were identified by seeking mediators that exhibited self-feedback. Though the data were segregated by outcome group prior to being subjected to DBN inference, the algorithm did not make assumptions about the connectivity of the network in any of the outcome sub-groups. Results of this analysis for each of the PALF sub-groups (as defined above) are shown in Fig. 2. First, similar to what we previously observed in non-survivors^9^, DBN inference suggested a primary network driven by a core motif consisting of HMGB1, which drives its own expression, in all patient sub-groups. However, certain differences were noted. In non-survivors, HMGB1 was the only mediator with an inferred positive self-feedback loop. In contrast, in the survivor sub-groups we observed, in addition to HMGB1, a cross-interaction consisting of MIG in APAPo + NAC, non-APAPo + NAC, and non-APAPo non-NAC, and IP-10 in non-APAPo non-NAC, each of which regulates its own expression.

### Dynamic Network Analysis (DyNA) of circulating inflammatory mediators shows differential trajectories that differentiate PALF patient sub-groups and suggests key network complexity thresholds

**Figure 3:**
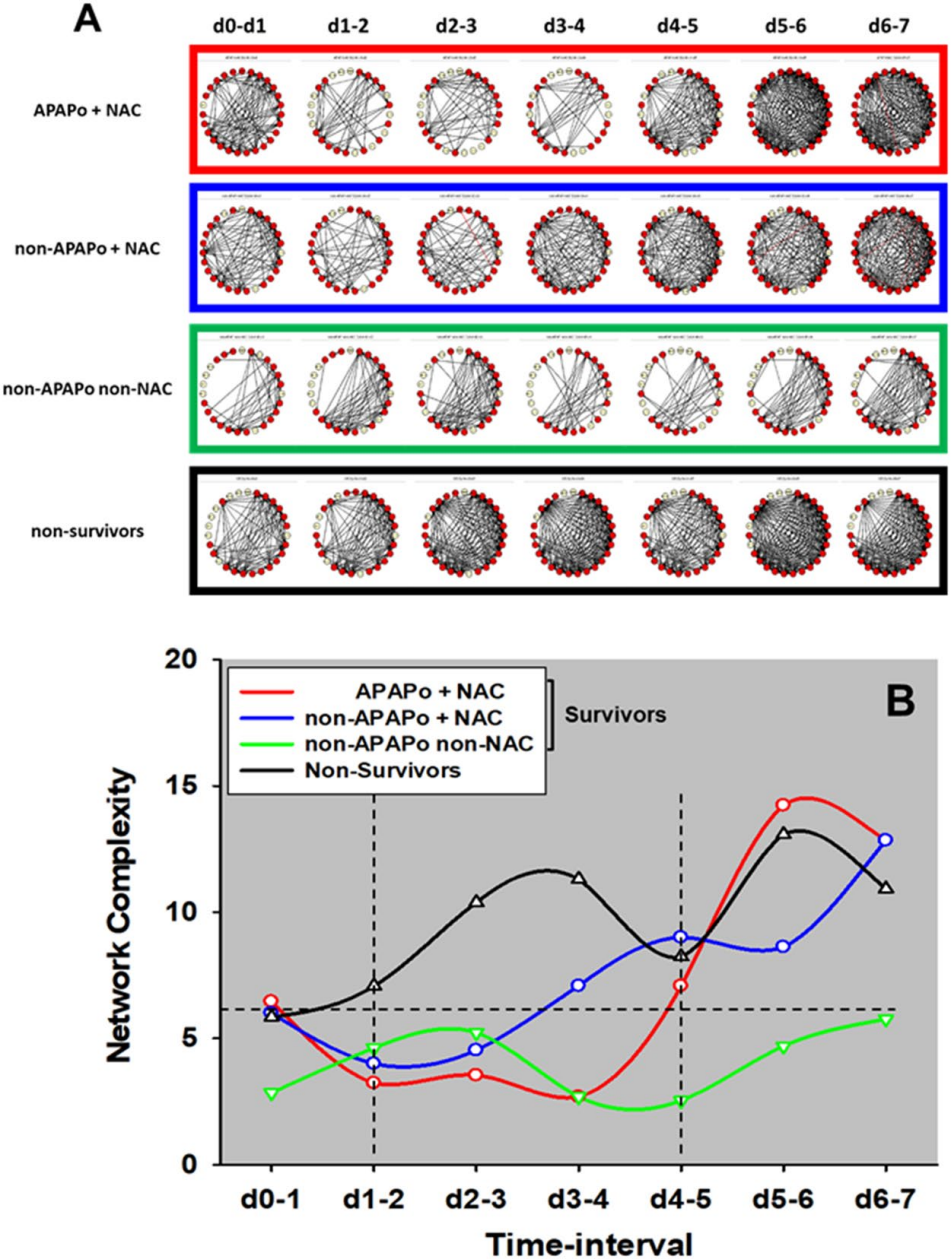
Dynamic Network Analysis (DyNA) of circulating inflammatory mediators in PALF patients. Circulating inflammatory mediators in serum samples from PALF spontaneous survivors were segregated into three sub-groups (APAPo + NAC [n = 13 patients], non-APAPo + NAC [n = 8 patients], and non-APAPo non-NAC [n = 40 patients]) and non-survivors (n = 12 patients). DyNA (stringency level = 0.7) was performed during each of the following seven time frames: d0-d1, d1–2, d2–3, d3–4, d4–5, d5–6, d6–7 as described in *Materials and Methods*. Panel A shows an overview of all the networks and mediator connections over all time intervals (the closed red circles represent mediators with at least one connection to another mediator, while open yellow circles represent mediators that had no connections to other mediators). Panel B shows the network complexity for the PALF sub-groups calculated as described in *Materials and Methods*.

Previously, we showed that PALF non-survivors had more robust dynamic networks of inflammation than those of survivors, in line with the concept of pathology driven by self-sustaining inflammation^9^. Accordingly, we next hypothesized that employing the same methodology would further differentiate among the three sub-groups of PALF survivors. DyNA^9,12,13^ was used to discern and compare the interconnections among inflammatory mediators in the three survivor sub-groups vs. non-survivors over defined ranges of time (a detailed DyNA output template is shown in Suppl. Fig. 2 for reference). In support of our hypothesis, DyNA suggested a different dynamic network connectivity in each of the patient sub-groups (Fig. 3). Comparison of these networks by total number of connections in each sub-group showed that all survivors had fewer connections (APAPo + NAC [651], non-APAPo + NAC [677], non-APAPo non-NAC [369]) as compared to non-survivors [869] (Fig. 3A). Furthermore, analysis of the network complexity suggested the presence of two threshold network complexity events: the first one at d1–2 following enrollment, and a latter one around d4–5 (Fig. 3B). Prior to the d1–2 threshold, the presence of similar complexity scores suggests similar dynamic inflammatory responses among the patient sub-groups. However, after the first hypothesized threshold, network complexity was clearly higher in non-survivors but not in the other patient sub-groups. After d4–5, the complexity trajectories of all sub-groups, except that of non-APAPo non-NAC patients, again became nearly undistinguishable from each other (Fig. 3B). The non-APAPo/non-NAC group of survivors exhibited the fewest network connections when initially entered into the study, and the fewest overall, with the least change over time. In this group, the complexity of the mediator interactions never rose above a threshold level of 5 at any time period, and these patients were coincidentally not administered NAC by the care team.

**Figure 4:**
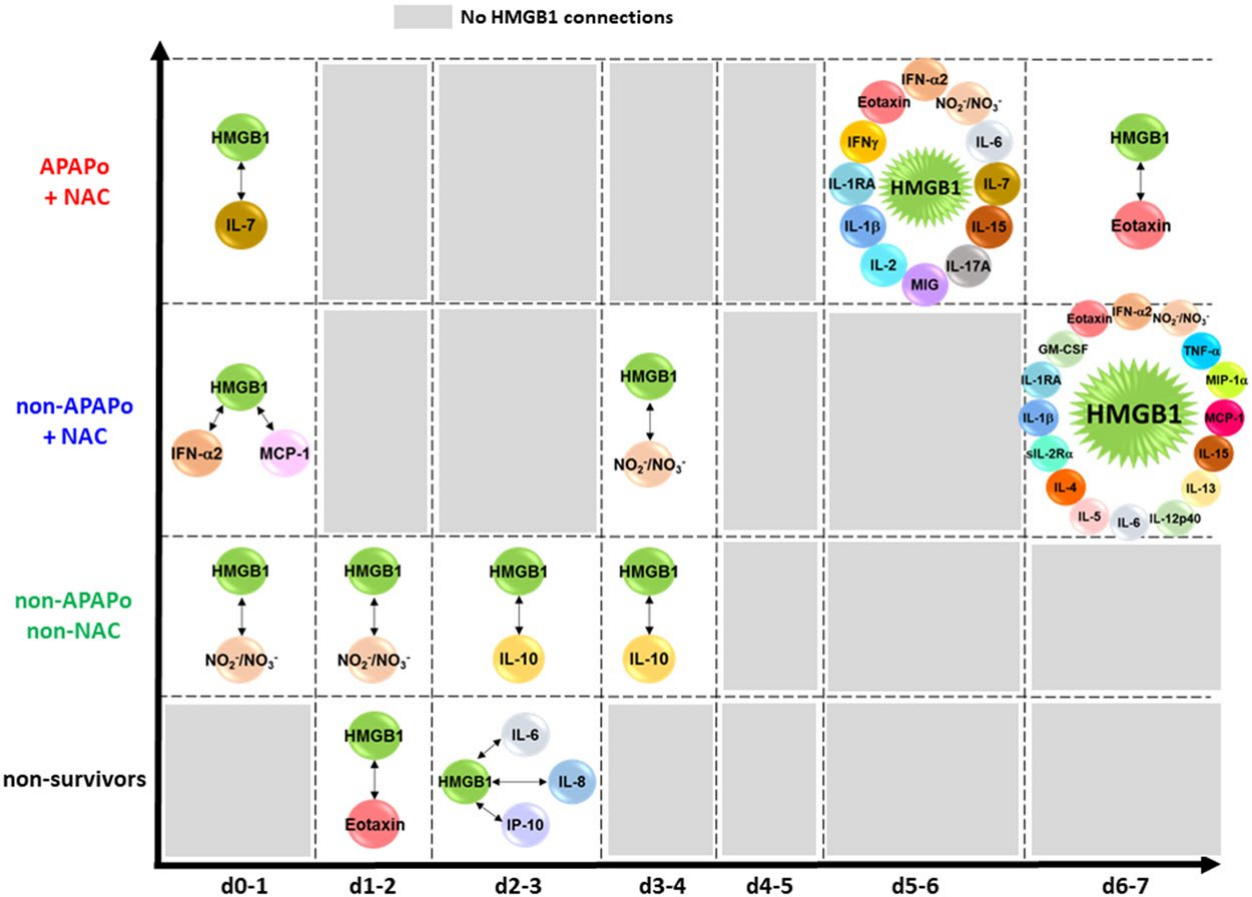
HMGB1 connectivity to other inflammatory mediators depends on the use of NAC in the context of APAPo in PALF. Circulating inflammatory mediators were assessed in serum samples from PALF spontaneous survivors (APAPo + NAC [n = 13 patients], non-APAPo + NAC [n = 8 patients], and non-APAPo non-NAC [n = 40 patients]) and non-survivors (n = 12 patients). DyNA was performed as described in Fig. 3. Figure panels highlight the significant connections of HMGB1, extracted from the dynamic patterns and overall network connectivity shown in Fig. 3A.

Based on our previous report^9^, as well as the implication of HMGB1 in the pathobiology of APAP toxicity in prior studies by others^14^, we hypothesized that the putative threshold events described above (Fig. 3B) are related to the differential HMGB1 connectivity in the different patient sub-groups. We therefore analyzed the DyNA networks associated solely with HMGB1. This analysis of connectivity in the HMGB1-focused DyNA showed that HMGB1 is most connected on d2–3 (connecting to IL-6, IL-8, and IP-10) in non-survivors, and has no connections at d3–4 and beyond (Fig. 4). In contrast, HMGB1 had no connections in the APAPo + NAC sub-group at d1–5. Furthermore, DyNA revealed a similar, relatively low number of HMGB1 connections in both the non-APAPo + NAC and non-APAPo non-NAC sub-groups at d0-d4 (Fig. 4). Interestingly, HMGB1 acquired additional connections between d5–7, but only in the APAPo + NAC and non-APAPo + NAC sub-groups. We note that comparison of the time-dependent changes in inflammatory mediators between PALF non-survivors with (n = 4 patients) and without NAC treatment (n = 8 patients) found no statistically significant difference in 26/27 of the inflammatory mediators assessed (data not shown), suggesting that the distinct and robust inflammatory networks in non-survivors are not dependent on the administration of NAC.

### Dynamic Network Analysis (DyNA) of inflammatory mediators in primary mouse hepatocytes confirms the central role for HMGB1 in the response to APAP toxicity

**Figure 5:**
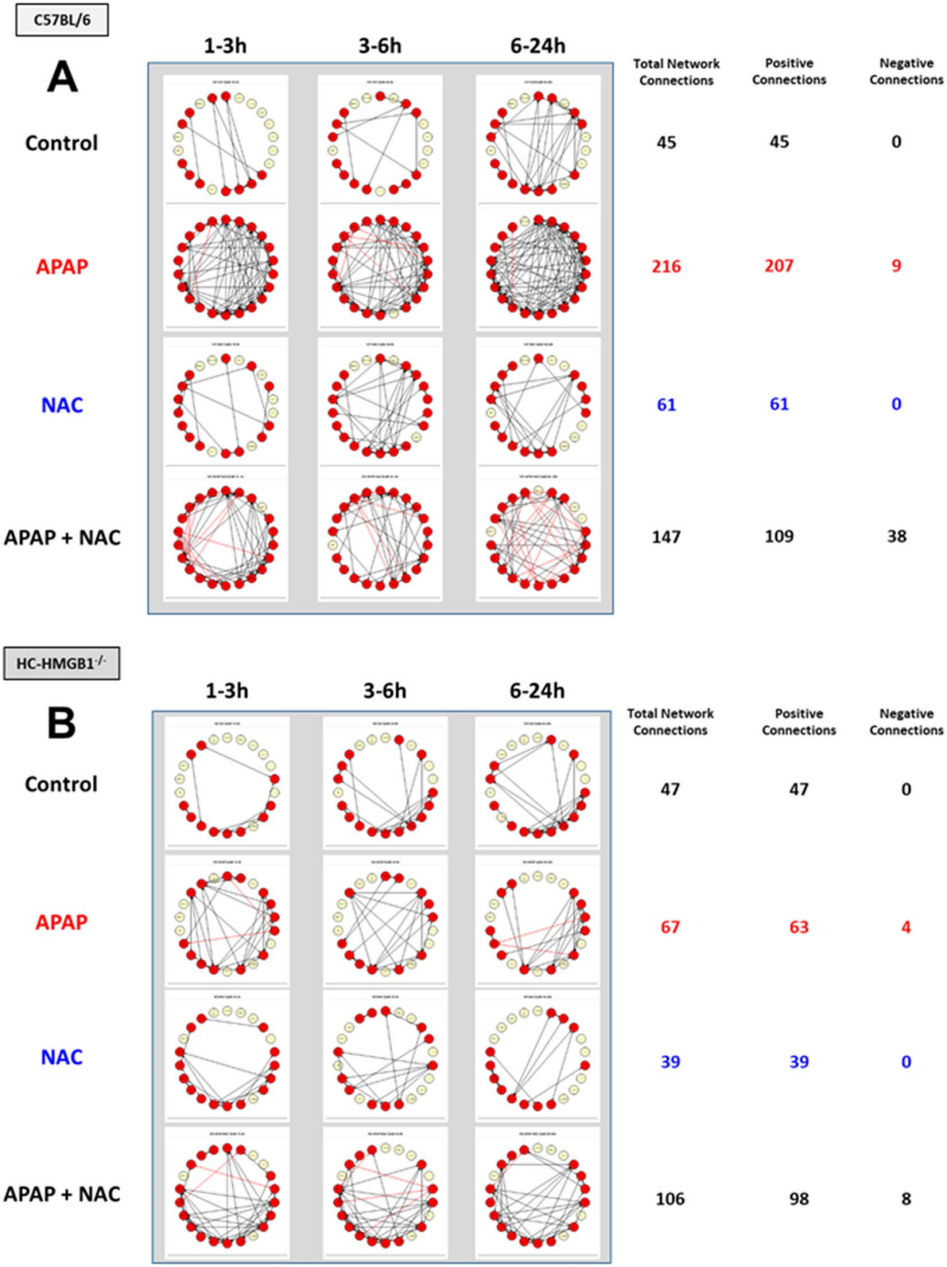
Dynamic Network Analysis (DyNA) of inflammatory mediators released by mouse HC from C57BL/6 and HC-HMGB1^−/−^ mice. Freshly isolated HC from C57BL/6 or HC-HMGB1^−/−^ mice were treated with 10 mM APAP alone (C57BL/6: n = 3, HC-HMGB1^−/−^: n = 5 mice) or for 1 h followed by 1 mM NAC (C57BL/6: n = 9, HC-HMGB1^−/−^: n = 6 mice) administered at 1–24 h following APAP (APAP + NAC) (C57BL/6: n = 3, HC-HMGB1^−/−^: n = 3 mice). Non-treated HC harvested at the same time points served as controls (C57BL/6: n = 12, HC-HMGB1^−/−^: n = 11 mice). Inflammatory mediators in supernatants were measured and analyzed using DyNA as described in *Materials and Methods*. An overview of all networks together with the number of network connections for each experimental condition (Control, APAP, NAC, and APAP + NAC) is shown in Panels A (C57BL/6 HC) and B (HC-HMGB1^−/−^ HC). Black and red arrows represent positive and negative connections, respectively.

**Figure 6:**
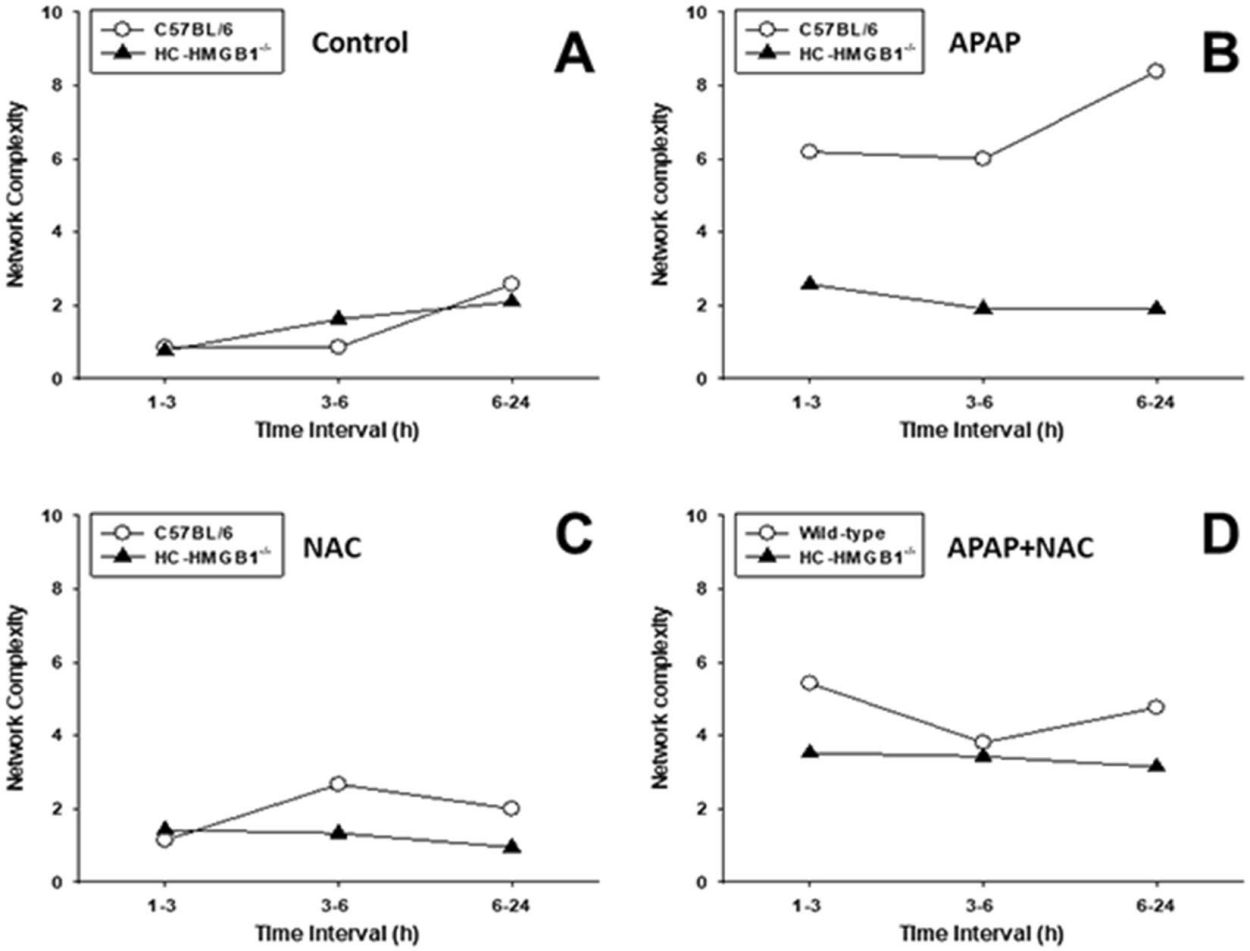
Inflammatory network complexity of mediators released by HC from C57BL/6 and HC-HMGB1^−/−^ mice suggests a central, coordinating role for HMGB1. Freshly isolated HC from C57BL/6 or HMGB1^−/−^ mice were incubated with 10 mM APAP alone or for 1 h followed by 1 mM NAC administered at 1–24 h following APAP as described in Fig. 5. Non-treated HC harvested at the time points served as controls. Inflammatory mediators in supernatants were measured and analyzed using DyNA as described in *Materials and Methods*. Comparison of inflammatory network complexity in C57BL/6 vs. HC-HMGB1^−/−^ HC in each experimental group is shown in Panel A (Control), Panel B (APAP), Panel C (NAC), and Panel D (APAP + NAC).

The effect of APAP in isolated cells has been studied widely and concentrations that range from 5 to 40 mM have been used to study APAP-mediated toxicity *in vitro*^15^. Given the central inflammatory role for HMGB1, we thought to compare the response to a toxic dose of APAP (10 mM) for 1 h alone or followed by 1 mM NAC added at 1, 3, 6 and 24 h following APAP in isolated mouse HC from C57BL/6 (wild-type) vs. HC-specific HMGB1-null mice (HC-HMGB1^−/−^). The time-courses of the 22 inflammatory mediators analyzed by Two-Way ANOVA showed different trajectories and revealed multiple inflammatory mediators that changed significantly (Suppl. Fig. 3). We next thought to compare the dynamic patterns of inflammatory mediators in isolated HC from C57BL/6 and HC-HMGB1^−/−^ mice under the four experimental conditions (untreated Control, APAP, NAC, APAP + NAC) using DyNA as described above. This analysis showed a much more complex network pattern and higher total number of network connections in C57BL/6 HC (Fig. 5A) as compared to HC-HMGB1^−/−^ cells (Fig. 5B). Interestingly, we also observed a difference in the number of negative mediator connections (mediators that change in an anti-correlated fashion in a given time-interval) depending on the use of NAC (APAP vs. APAP + NAC) and mouse strain: while in C57BL/6 HC the presence of negative connections increased from 4.2% (9/216 in APAP) to 25.9% (38/147 in APAP + NAC) (Fig. 5A), in HC-HMGB1^−/−^ the number of negative connections remained essentially unchanged (6% vs. 7.5% in APAP [4/67/] and APAP + NAC [8/106], respectively) **(**Fig. 5B). Furthermore, the quantification of network complexity revealed a striking difference in the response of HC to APAP from the two mouse strains. There was no difference in control (Fig. 6A), NAC alone (Fig. 6C), or APAP + NAC-treated cells (Fig. 6D). However, APAP-treated C57BL/6 HC exhibited a much more complex dynamic networks than HC-HMGB1^−/−^ cells (Fig. 6B), suggesting that HMGB1 plays a central role in orchestrating the inflammatory response to APAP.

## Discussion

Pediatric Acute Liver Failure is a rare but life-threatening clinical syndrome whose onset is not completely understood^1^. Despite recent advances in diagnosis and management, specific diagnostic and therapeutic targets and tools to predict spontaneous survival/death or to inform liver transplantation decisions in PALF are still not available. The present study sought to define central regulatory mechanisms associated with APAP-induced inflammation in PALF. We have reported previously that computational and data-driven analysis of principal inflammatory drivers and dynamic inflammatory networks could discriminate among sub-groups of PALF patients^9^. As an extension of these previous studies^8,9^, we hypothesized that novel insights into inflammatory responses in APAPo + NAC, non-APAPo + NAC, and non-APAPo non-NAC could be defined and differentiated from that of PALF non-survivors using multiplex assays of inflammatory mediators and data-driven analysis. The key insights from the present study are (1) the identification of a putative temporal threshold of inflammatory network connectivity; (2) the demonstration of a central role for HMGB1 in nucleating systemic inflammatory responses in PALF; and (3) a demonstration of the translational potential of combining *in vitro*, *in silico*, and clinical studies.

Numerous studies, both *in vitro* and *in vivo*, have been directed to investigate the role of HC in the context of Acute Liver Failure (ALF), but to a lesser extent in PALF. Of particular interest to our present work are those experimental and clinical studies related to APAP overdose, the leading cause of ALF in most industrialized countries^16^. Those studies include the involvement of critical signaling pathways (e.g. APAP-mediated induction of receptor-interacting protein (RIP) signaling as a critical switch for hepatocellular necrosis^17^); the identification of proteins as potential biomarkers of liver injury (e.g. high levels of sFas and hepatocyte growth factor (HGF) in APAP-related ALF^18^; associations between levels of given proteins (e.g of M-30 antigen and CPS1) with clinical outcomes^18,19^; and increased levels of HMGB1 in both adult ALF^20^ and PALF^8,9^. We now suggest that the network coordination of HMGB1 is a key aspect of HC biology in the context PALF-associated inflammation.

A recent study from the PALF Study Group has shown that children with undetermined diagnosis have lower spontaneous survival and higher rates of transplantation and death than other diagnostic groups, suggesting that reducing systemic inflammation would also limit neuroinflammation and improve survival significantly^21^. We have previously shown that PALF non-survivors had more robust dynamic networks of inflammation than those of survivors^9^, in line with the concept of pathology driven by self-sustaining inflammation in other disease states^22^. Dynamic Network Analysis^9,12,13^ highlighted the dynamic differences in the interconnections among inflammatory mediators in the three survivor sub-groups vs. non-survivors, and in particular the different network connectivity and total number of connections in each of the patient sub-groups (NS > APAPo + NAC, non-APAPo + NAC, non-APAPo non-NAC). This analysis, in line with our previous report^9^ and other studies implicating HMGB1 in the pathobiology of APAP toxicity^14^, also suggested the presence of two threshold network complexity events that seemed to be related to the differential HMGB1 connectivity in the different patient sub-groups.

The current approach to the management of APAP toxicity continues to be limited by imprecise and time-constrained risk assessments and late-stage markers of liver injury. Our present results suggest that the connectivity of HMGB1 may be a potential novel biomarker or drug target for PALF. Our results suggest that HMGB1 inflammatory connectivity, is affected significantly by the differential use of NAC in the context of APAP overdose. N-acetylcysteine has been the most effective therapy against APAP-induced liver injury in both adults^3,23^ and children^2,24^. This treatment is effective in patients with acute APAP-induced ALF, but its use for treating patients with liver injury caused by chronic APAP exposure warrants further investigation^2^. For example, a retrospective study of pediatric non-APAP-induced ALF showed that NAC was associated with a shorter length of hospital stay, higher incidence of native liver recovery without transplantation, and better survival after transplantation^25^. In contrast, the use of NAC failed to improve 1-year transplant-free survival in children with non-APAP ALF, highlighting the importance of conducting prospective pediatric drug trials, regardless of results in adults^4^.

A practical and ethical consideration that complicates predictive biomarker research in this area is the clinical need to deliver antidote treatment within 10 h of APAP overdose^26^. In adult liver injury, inclusion of HMGB1 in a biomarker panel has been suggested to improve the speed of clinical decision-making, both in the treatment of acute liver injury and patient-individualized treatment strategies^27^. Our analysis suggests that the patients who were coincidentally not administered NAC by the care team (non-APAPo non-NAC group) exhibited both the fewest overall inflammatory network connections and the fewest connections when entered initially into the study. Interestingly, HMGB1 appeared to reemerge on day 5–7 in the APAPo + NAC and non-APAPo + NAC cohorts. HMGB1 has been shown to promote wound healing and endogenous stem cell differentiation^28^. Thus, HMGB1 may play a role not only in the initiation and propagation of the inflammatory response, but also in hepatocellular regeneration following liver injury.

Unraveling the association of HMGB1 network connectivity is difficult in the clinical setting. Accordingly, we reasoned that the putative role of HMGB1 in coordinating the hepatic inflammatory response in PALF patients could be recapitulated *in vitro*. Ordinarily, extrapolating directly from *in vitro* studies to the clinical setting would be difficult if not impossible. However, the use of advanced computational tools demonstrated a qualitative agreement at the network level between mouse HC exposed to APAP + NAC *in vitro* vs. APAPo + NAC patients. Thus, we tested the hypothesis that a reduction or elimination of HMGB1 in HC *in vitro* would block the APAP-induced evolution of dynamic networks of inflammation. In support of this hypothesis, we found a much more complex network pattern and higher total number of network connections in C57BL/6 HC as compared to HC-HMGB1^−/−^ cells, suggesting that indeed HMGB1 plays a central role in orchestrating the inflammatory response to APAP. This analysis was performed in the absence of adjacent immune cells that would be present *in vivo*, and therefore likely underestimates the full magnitude and complexity of the HMGB1-driven inflammatory response induced be HMGB1 following APAP toxicity. HMGB1 is a ubiquitous nuclear protein present in almost all cell types with both intracellular functions in the nucleus and extracellular functions as a DAMP^29^. In the context of liver injury, neutralizing HMGB1was protective against APAP-induced inflammation in mice^6^. Interestingly, in our experiments the levels of HMGB1 were significantly reduced in APAP-treated HC-HMGB1^−/−^ cells, yet some form of HMGB1 or HMGB1 reactive molecule was detected in the supernatants of those cultures. This may be due to differential processing of HMGB1, or to a small contamination with non-HC cells in our primary cultures. Hyperacetylation of HMGB1 shifts its equilibrium from a predominant nuclear location toward a cytosolic and subsequent extracellular presence^29^. In addition, it has been demonstrated that the extracellular activity of HMGB1 as inflammatory mediator is closely related to the redox state of its three key cysteine residues^30,31^. Under strong oxidizing conditions such as those seen with APAP exposure *in vitro*, the oxidation of some or all of the cysteine residues could lead to loss of biological activity of HMGB1^30^. Future studies will need to address the potential role of these HMGB1 modifications in coordinating networks of inflammation. Nonetheless, in our system the overall effect of APAP is likely not due to the presence of a pro-inflammatory form of HMGB1, as evidenced by the distinct and low network complexity in HC-HMGB1^−/−^ cells as compared to wild-type cells.

Similar to our previous PALF studies^8,9^, there are some unavoidable limitations to the present work. First, we are unable to determine the onset for the systemic inflammation in PALF patients as the exact onset date for PALF cannot be determined. Equally important is that fact we only analyzed 8 days of samples, so changes in dynamic networks either before or after this time period remain unidentified; future studies will address this issue. The same applies to samples from participants with short, mild events or severe, rapid progression to death or transplantation that were excluded from this analysis given the analytical requirement for at least three available blood samples. We also note the relatively small number of patients and age heterogeneity in the non-survivors group. Finally, we acknowledge the difficulties in both measuring all the potential inflammatory mediators involved in (and relevant to) PALF and translating these complex analyses and hypotheses into a diagnostic test that could be used to assess outcome.

In conclusion, our results suggest that the inflammatory dynamic networks, and in particular HMGB1 connectivity, are dependent on (or reflect) the use of NAC in the context of APAP toxicity in PALF patients. In isolated HC, high APAP dose led to a much more complex dynamic networks in C57BL/6 HC than in cells from HMGB1^−/−^ animals, suggesting that HMGB1 plays a central role in orchestrating the inflammatory response to APAP. In a broader context, our findings extend previous observations and suggest that analysis of dynamic networks of inflammation may be used to differentiate patient etiologies, which, when combined with appropriate *in vitro* studies, may lead to novel therapeutic opportunities for PALF. Specifically, PALF patients with high HMGB1 levels and high levels of inflammatory mediators early could be considered for therapies that target HMGB1, as they are at the greatest risk for death.

## Methods

### Selection of PALF patients and study design

This is a multi-center study conducted through the Pediatric Acute Liver Failure Consortia (PALF; National Institutes of Health/National Institutes of Diabetes, Digestive, and Kidney Disease: 5U01 DK072146). The study was performed in accordance with the relevant guidelines and regulations and was approved by the Institutional Review Boards from all participating institutions (listed in the Acknowledgments), with written informed consent from parents and/or legal guardians and Certificate of Confidentiality provided by NIH. Entry criteria for the study included children less than 18 years of age with (1) no known evidence of chronic liver disease, (2) biochemical evidence of acute liver injury, and (3) hepatic-based coagulopathy (not corrected with parenteral vitamin K) defined as a prothrombin time (PT) ≥ 15 seconds or international normalized ratio (INR) ≥ 1.5 in the presence of clinical hepatic encephalopathy (HE), or a PT ≥ 20 seconds or INR ≥ 2.0 regardless of the presence or absence of HE. After enrollment, demographic and clinical data were recorded daily for up to seven days with a single daily serum sample for research scheduled to be collected on the calendar day of enrollment (d0) or with the first morning blood draw following enrollment and daily for up to seven days (d1–d7), or until death, LTx, or discharge from hospital. Serum samples were promptly frozen at −80 °C at the enrollment site and later batch-shipped to the research bio-repository for long-term storage. At the time of this analysis, PALF study enrollment included 1,144 participants and the selection criteria for the analysis is shown in Fig. 1. Participants were selected if they had at least three daily samples with at least 100 µl of serum available. Selection criteria were met by 73 participants and clinical outcomes at twenty-one days following enrollment were assigned as follows: survival without LTx (spontaneous survivors, n = 61 patients) or death without LTx (non-survivors, n = 12 patients). Those receiving LTx prior to 21 days following enrollment were excluded. Otherwise, our cohort would have been a mixed cohort of participants who would have lived or would have died within 21 days had LTx not interrupted the clinical course. In the APAPo cohort, all received NAC and were survivors. We note that given the clinical nature of PALF, the interval between time of ingestion, recognition/acknowledgement of ingestion, admission to hospital, and enrollment cannot be expected to be uniform for all APAP PALF participants. In a previous study^32^, we reported that among 58 PALF cases with acute APAP toxicity, participants were enrolled between 1 to 6 days following ingestion with 93% enrolled within <4 days. Examining PALF survivors who did and did not receive NAC may provide insight into the impact of NAC in non-APAP PALF. In our previous study, we examined the cohort of spontaneous survivors as a whole^9^. For this new analysis, survivors were divided into three sub-groups including participants in a placebo-controlled clinical trial of NAC^4^: APAPo plus NAC, non-APAPo plus NAC, and non-APAPo non-NAC (Table 1). PALF non-survivors were a heterogeneous group that included 4 patients who received NAC and served to characterize differences in their inflammatory network from the three sub-groups of survivors. Serum samples from participants meeting all criteria were shipped from the NIH biorepository to the Surgery Research Laboratory at the University of Pittsburgh for Luminex™ analysis (see below). A comprehensive table with detailed demographic and clinical data in both survivors and non-survivors can be found in a previous publication from our group^9^.

**Table 1:** Age distribution for PALF Study Patients.

Outcome	Patient Sub-group	No. of patients	Age (mean)	Age (median)	Q1–Q3
survivors	APAPo + NAC	13	15.2	16.1	15.3–17.7
	non-APAPo + NAC	8	11.8	13.1	9.0–15.6
	non-APAPo non-NAC	40	5.5	3.1	1.1–9.7
	All survivors	61	8.4	6.7	1.5–15.3
non-survivors	—	12	3.4	0.1	0.0–5.3

### Mouse hepatocyte isolation and culture

All experiments involving animals were performed in accordance with relevant NIH guidelines and regulations and were approved by the Animal Care and Use Committee of the University of Pittsburgh. Primary mouse hepatocytes (HC) were harvested from male wild-type C57BL/6 mice (purchased from Charles River Laboratories, Wilmington, MA) and hepatocyte-specific HMGB1-null mice (HC-HMGB1^−/−^, generated on a C57BL/6 genetic background^33^) and plated as previously described^13^. Briefly, after overnight incubation, the medium was removed and cell monolayers (200,000 cells/well) were further incubated with fresh media containing 5% heat-inactivated calf serum and 10 mM Acetaminophen (APAP) alone or for 1 h followed by 1 mM N-acetylcysteine (NAC) administered at multiple time-points from 1 to 24 h following APAP (APAP + NAC) (n = 3 independent experiments performed in triplicate or as otherwise indicated in the Figure Legends). At the end of each experiment, the cell supernatants were stored at −20 °C until further analysis (see below). APAP was purchased from Sigma-Aldrich (St Louis, MO) and dilutions were made using a 250 mM stock solution prepared in ethanol. NAC was purchased from Sigma-Aldrich (St Louis, MO) and dilutions were made in cell culture media. Non-treated HC and HC treated with NAC alone for the times indicated served as control.

### Assays of inflammatory mediators

We measured a number of cytokines and chemokines that serve as biomarkers for the complex inflammatory response using the Luminex™ 100 IS system (Luminex™, Austin, TX) and the Human 25-plex^®^ Luminex™ and Milliplex^TM^ Mouse Cytokine/Chemokine Panel I beadsets (Millipore, Billerica, MA). These antibody bead kits include:

Human (25 mediators): Eotaxin, Granulocyte-Macrophage Colony-Stimulating Factor (GM-CSF), Interferon (IFN)-α2, IFN-γ, Interleukin (IL)-1β, IL-1 Receptor Antagonist (IL-1RA), IL-2, Soluble IL-2 Receptor α chain (sIL-2Rα), IL-4, IL-5, IL-6, IL-7, IL-8, IL-10, IL-12p40, IL-12p70, IL-13, IL-15, IL-17A, IFN-γ-inducible Protein of 10 kDa (IP-10; CXCL10), Monocyte Chemotactic Protein-1 (MCP-1; CCL2), Monokine Induced by γ-Interferon (MIG; CXCL9), Macrophage Inflammatory Protein (MIP)-1α, MIP-1β, and Tumor Necrosis Factor (TNF)-α).

Mouse (20 mediators): GM-CSF, IFN-γ, IL-1α, IL-1β, IL-2, IL-4, IL-5, IL-6, IL-10, IL-12p40, IL-12p70, IL-13, IL-17, IP-10/CXCL10, Keratinocyte-derived Cytokine (KC/CXCL1), MCP-1/CCL2, MIG/CXCL9, MIP-1α/CCL3, TNF-α, and Vascular Endothelial Growth Factor (VEGF).

In addition, all samples were assayed for HMGB1 using a commercially available ELISA (Shino-Test, Kanagawa, Japan). We found that the cell culture media supplemented with calf serum used in our study contained significant amounts of HMGB1 or HMGB1-like protein as detected by the ELISA kit. Therefore, the concentrations of HMGB1 released by mouse HC were corrected before the computational analyses and are shown in Suppl. Fig. 3. The nitric oxide (NO) reaction products NO_2_^−^ + NO_3_^−^ were assayed using the nitrate reductase method (Cayman Chemical, Ann Arbor, MI). All mediator data were measured in pg/ml except for NO_2_^−^ + NO_3_^−^ measured in µM. The time courses of the inflammatory mediators were then plotted, shown as box plots representing the 25^th^ and 75^th^ percentiles with a line at the median and error bars defining the 10^th^ and 90^th^ percentile in PALF patients, and as mean ± SEM in mouse HC experiments.

### Statistical and data-driven computational analysis

Two-Way Analysis of Variance (ANOVA) followed by the Holm-Sidak post-hoc test was used to analyze the response and time-dependent changes in inflammatory mediators across PALF sub-groups and in the mouse HC experiments using SigmaPlot (Systat Software, San Jose, CA) as indicated.

Dynamic Bayesian Network (DBN) inference was carried out in order to define the most likely single network structure that best characterizes the dynamic interactions among systemic inflammatory mediators across all time points and, in the process, suggesting likely feedback structures that define central nodes. The networks may suggest possible mechanisms by which the progression of the inflammatory response differs within a given patient sub-group. Characterization of central nodes serves to suggest possible control points for a given dynamic process. In this analysis, time courses of unprocessed inflammatory mediator measurements from each patient were used as input for a DBN inference algorithm, implemented in MATLAB® as described previously for gene array data^34^ and modified by our group for the study of systemic acute inflammation^8,9,35^.

Dynamic Network Analysis (DyNA) was carried out to define, in a granular fashion, the central inflammatory network nodes as a function of both time and PALF patient sub-group, and experimental condition *in vitro*. Using inflammatory mediator measurements of at least three time-points for each patient, networks were created over seven consecutive time periods (d0-d1, d1–2, d2–3, d3–4, d4–5, d5–6, d6–7) using MATLAB® software^8,9,13^. Connections, defined as the number of trajectories of serum inflammatory mediators that move in parallel, were created if the Pearson correlation coefficient between any two nodes (inflammatory mediators) at the same time-interval was greater or equal to a threshold of 0.7 (a correlation value commonly used to characterize trajectories that move in parallel either up or down). The network complexity for each time-interval was calculated using the following formula: Sum (N_1_ + N_2_ + … + Nn)/n-1, where N represents the number of connections for each mediator and n is the total number of mediators analyzed. The total number of network connections represents the sum of the number of connections across all time-intervals for all patients in a given sub-group. The same methodology was applied to create networks using inflammatory mediator measurements over three consecutive time periods (1–3 h, 3–6 h, 6–24 h) from mouse HC as described.

## Supplementary information

Suppl. Info

Luminex data

## Data Availability

All data generated or analyzed during this study are included in this published article (and its Supplementary Information files).
