# Supplementary material for: HMGB1 is a Central Driver of Dynamic Pro-inflammatory Networks in Pediatric Acute Liver Failure induced by Acetaminophen
Source: Sci Rep. 2019 Apr 12;9:5971. doi: 10.1038/s41598-019-42564-5 (PMC6461628; doi:10.1038/s41598-019-42564-5)
Supplement: Supplementary file 1 — Suppl. Info [file 41598_2019_42564_MOESM1_ESM.pdf]

HMGB1 is a Central Driver of Dynamic Pro-inflammatory Networks in Pediatric Acute Liver Failure induced by Acetaminophen

Ruben Zamora[1,2], Derek Barclay[1], Jinling Yin[1], Estella M. Alonso[3], Mike A. Leonis[4], Qi Mi[1], Timothy R. Billiar[1,2], Richard L. Simmons[1], Robert H. Squires[5], Yoram Vodovotz[1,2,*]

[1]Department of Surgery, University of Pittsburgh, Pittsburgh, PA 15213

[2]Center for Inflammation and Regenerative Modeling, McGowan Institute for Regenerative Medicine, Pittsburgh, PA 15219

[3]Ann & Robert H. Lurie Children's Hospital of Chicago, Chicago, IL 60611

[4]Department of Pediatrics, University of Alabama at Birmingham, Birmingham, AL 35233.

[5]Department of Pediatrics, University of Pittsburgh, Pittsburgh, PA 15213

Supplementary Figures

Fig. S1. Time-dependent release of inflammatory mediators in PALF patients. Serum samples from PALF spontaneous survivors were assessed for inflammatory mediators and segregated into three sub-groups (APAPo+NAC [n=13 patients], non-APAPo+NAC [n=8 patients], and non-APAPo non-NAC [n=40 patients]) and non-survivors (n=12 patients). Box plots represent the 25th and 75th percentiles with a line at the median and error bars defining the 10th and 90th percentiles (significance level set at $P<0.05$, analyzed by Two-Way ANOVA followed by Holm-Sidak *post hoc t*est).

Fig. S2. Explanation of DyNA output. A representative DyNA output showing a network of inflammatory mediator connections over a specific time interval. The closed red circles (nodes) represent mediators with at least one connection to another mediator, while open yellow nodes represent mediators with no connections to other mediators. Black lines (edges) represent positive connections linking mediators that move in the same direction, while red edges represent negative connections that link mediators that move in opposite direction.

Fig. S3. Time-dependent release of inflammatory mediators by HC from C57BL/6 and HC-HMGB1$^{-/-}$ mice. Analysis of supernatant concentrations of inflammatory mediators released by freshly isolated primary HC from C57BL/6 and HC-HMGB1$^{-/-}$ mice. Cells were treated with 10 mM APAP alone (C57BL/6: n=3, HMGB1$^{-/-}$: n=5 mice) or for 1h followed by 1 mM N-acetylcysteine (NAC) (C57BL/6: n=9, HMGB1$^{-/-}$: n=6 mice) administered at 1-24h following APAP (APAP+NAC) (C57BL/6: n=3, HMGB1$^{-/-}$: n=3 mice). Non-treated HC harvested at the same time points served as controls (C57BL/6: n=12, HMGB1$^{-/-}$: n=11 mice). Results are presented as the mean ± SEM (significance level set at $P<0.05$, analyzed by Two-Way ANOVA followed by Holm-Sidak *post hoc* test).

Suppl. Fig. 1

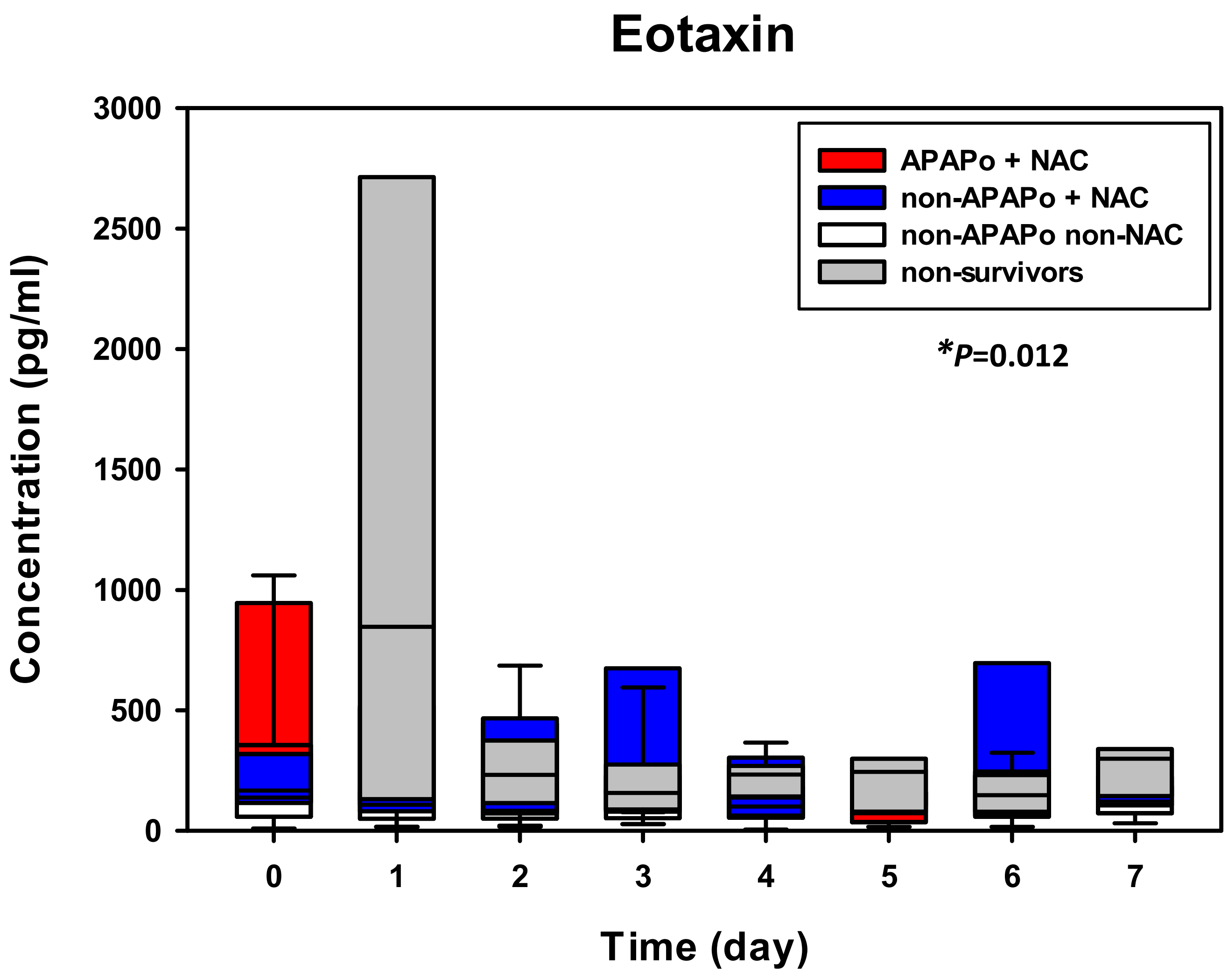

Eotaxin
Concentration (pg/ml)
3000
2500
2000
1500
1000
500
0
APAPo + NAC
non-APAPo + NAC
non-APAPo non-NAC
non-survivors
*P=0.012
0
1
2
3
4
5
6
7
Time (day)

Suppl. Fig. 1

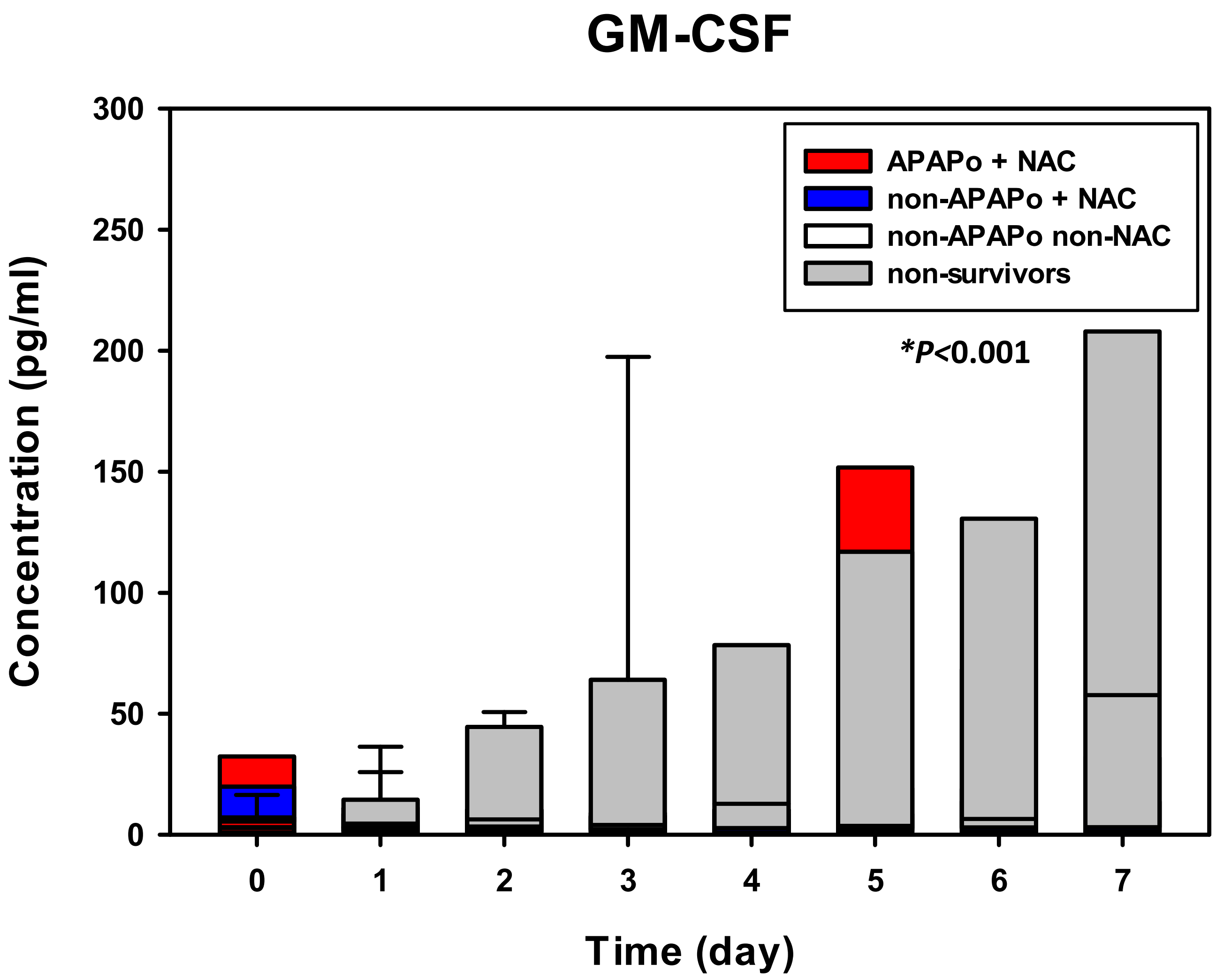
GM-CSF
APAPo + NAC
non-APAPo + NAC
non-APAPo non-NAC
non-survivors
*P<0.001
Concentration (pg/ml)
300
250
200
150
100
50
0
0
1
2
3
4
5
6
7
Time (day)

Suppl. Fig. 1

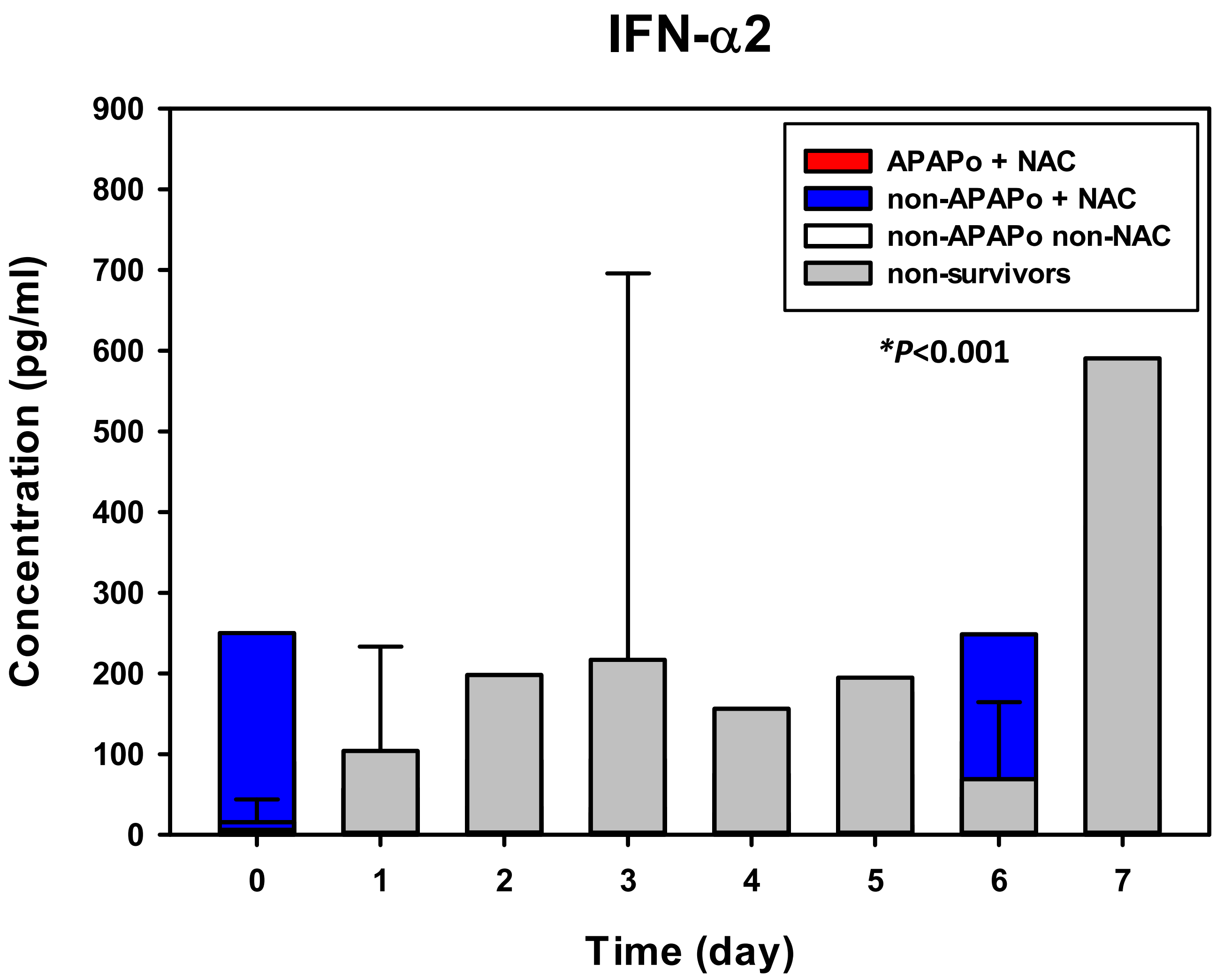
IFN-α2
Concentration (pg/ml)
900
800
700
600
500
400
300
200
100
0
0
1
2
3
4
5
6
7
Time (day)
APAPo + NAC
non-APAPo + NAC
non-APAPo non-NAC
non-survivors
*P<0.001

Suppl. Fig. 1

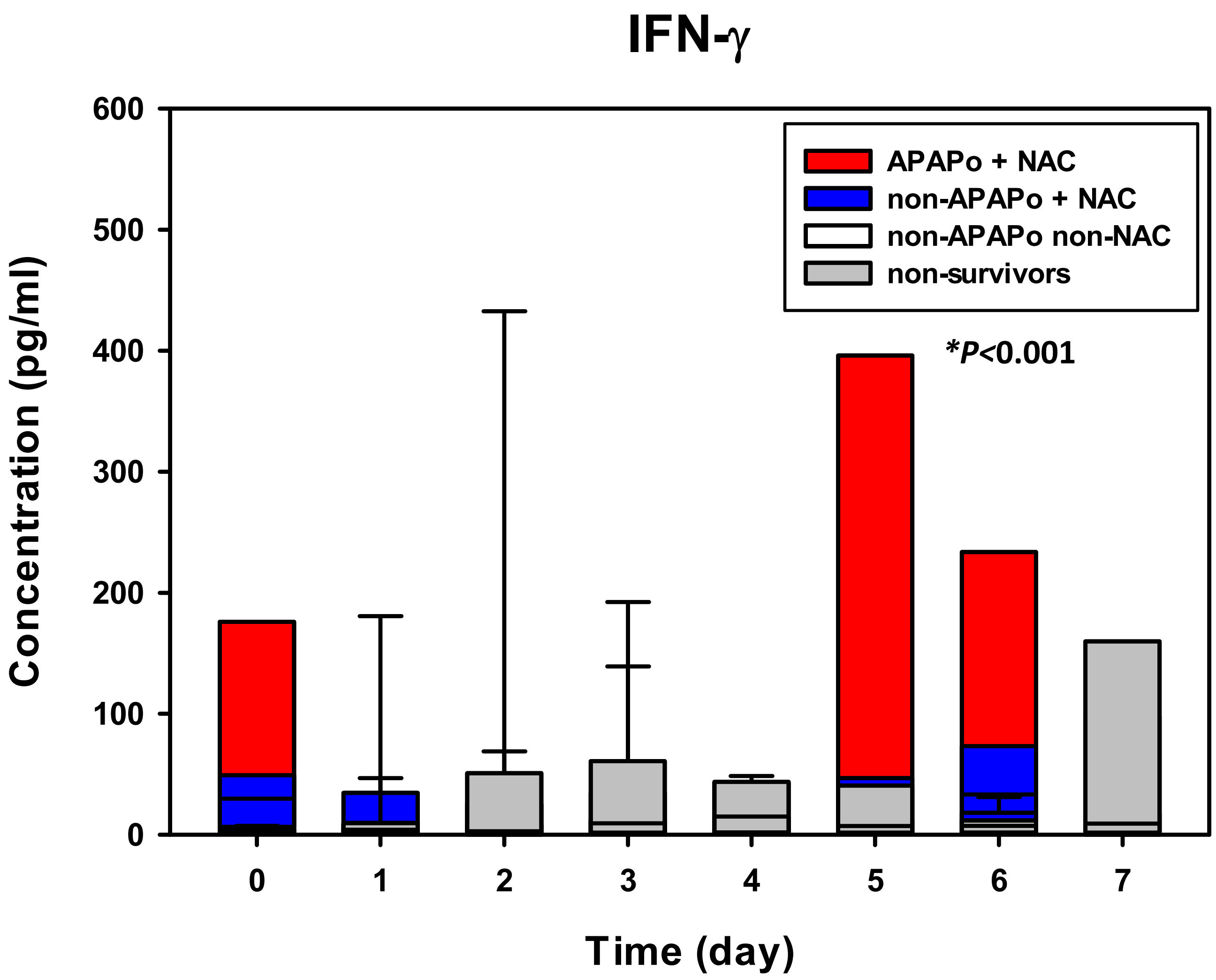

IFN-γ
600
500
400
300
200
100
0
Concentration (pg/ml)
APAPo + NAC
non-APAPo + NAC
non-APAPo non-NAC
non-survivors
*P<0.001
0
1
2
3
4
5
6
7
Time (day)

Suppl. Fig. 1

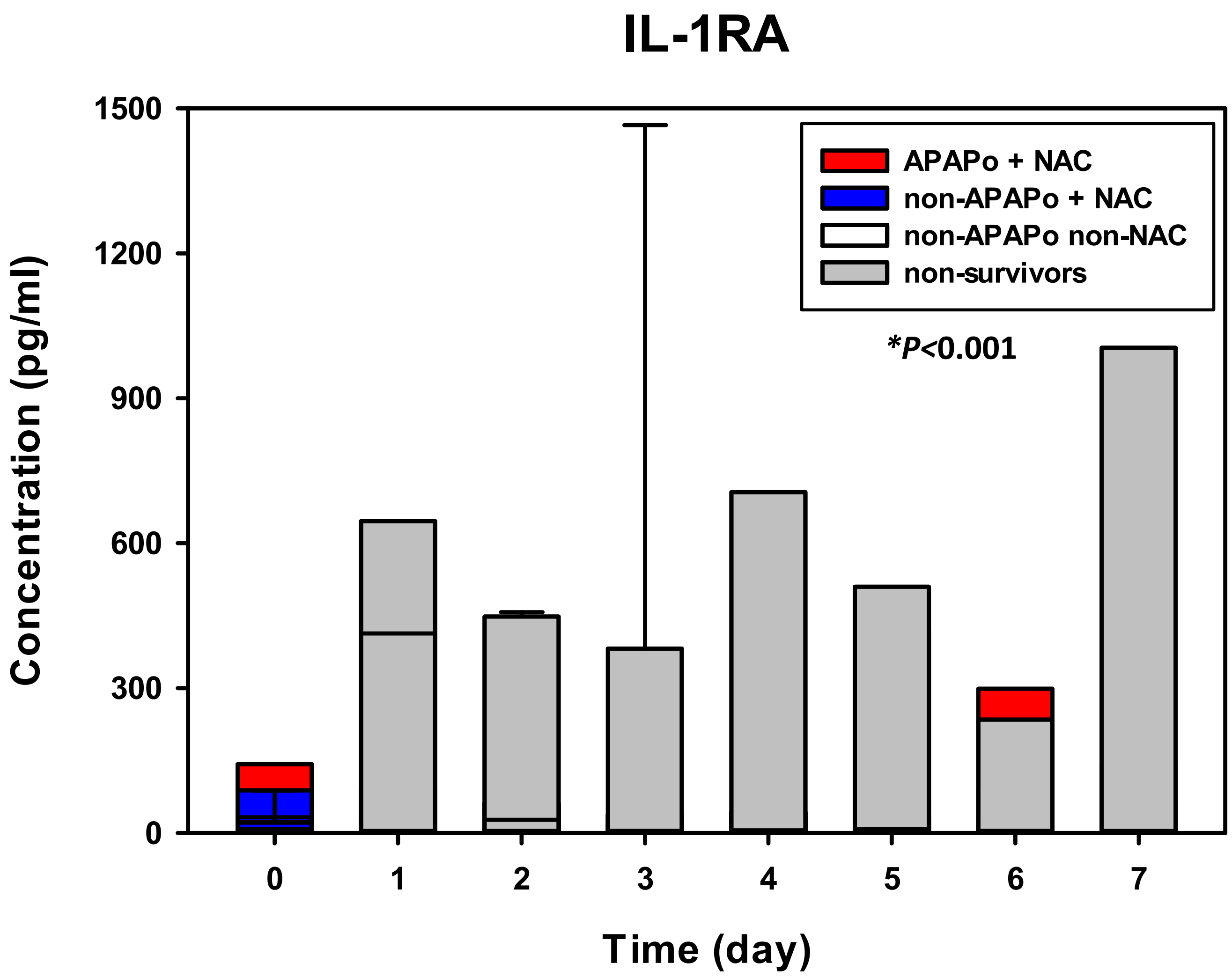
IL-1RA
1500
1200
900
600
300
0
Concentration (pg/ml)
APAPo + NAC
non-APAPo + NAC
non-APAPo non-NAC
non-survivors
*P<0.001
0
1
2
3
4
5
6
7
Time (day)

Suppl. Fig. 1

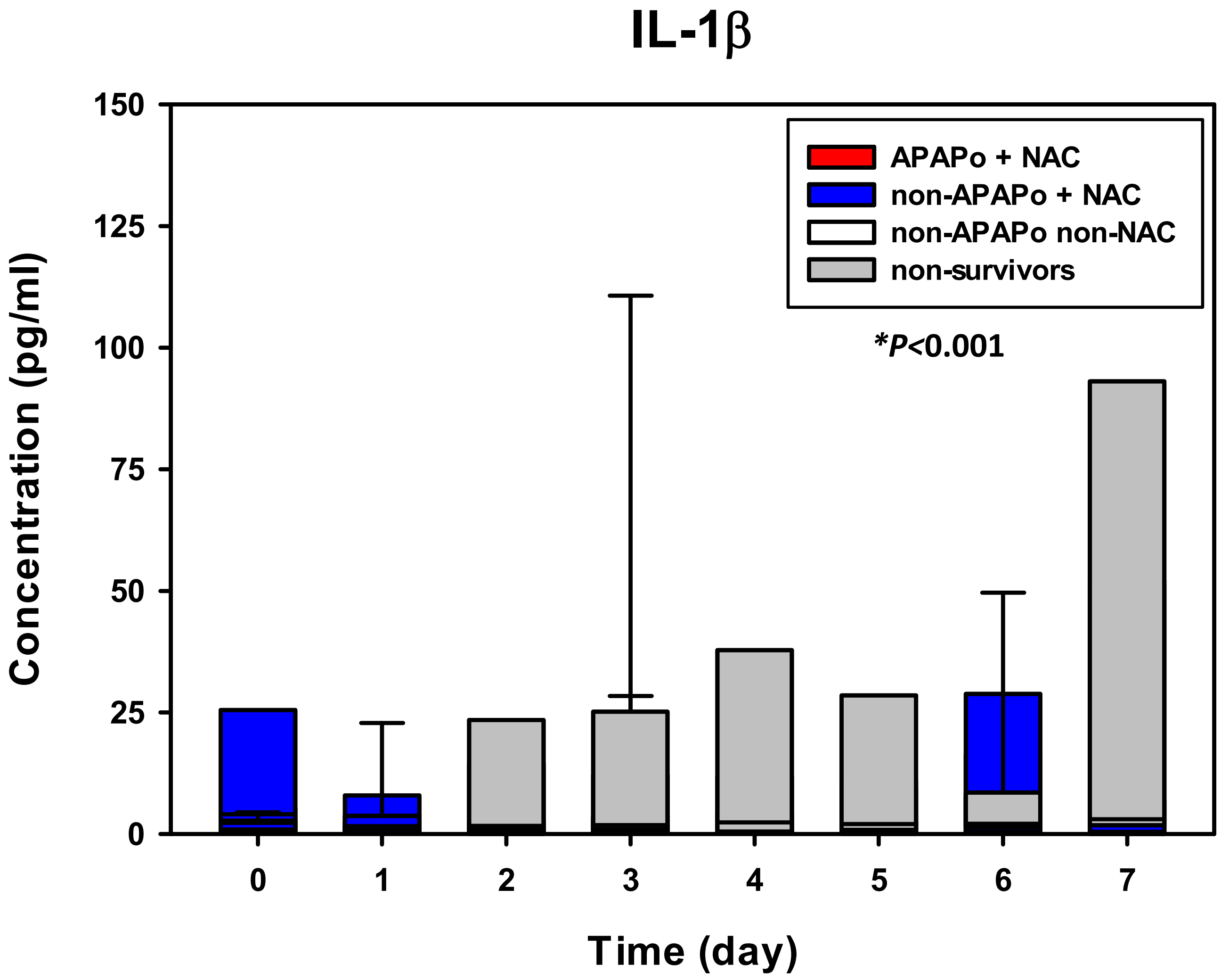
IL-1β
Concentration (pg/ml)
150
125
100
75
50
25
0
APAPo + NAC
non-APAPo + NAC
non-APAPo non-NAC
non-survivors
*P<0.001
0
1
2
3
4
5
6
7
Time (day)

Suppl. Fig. 1

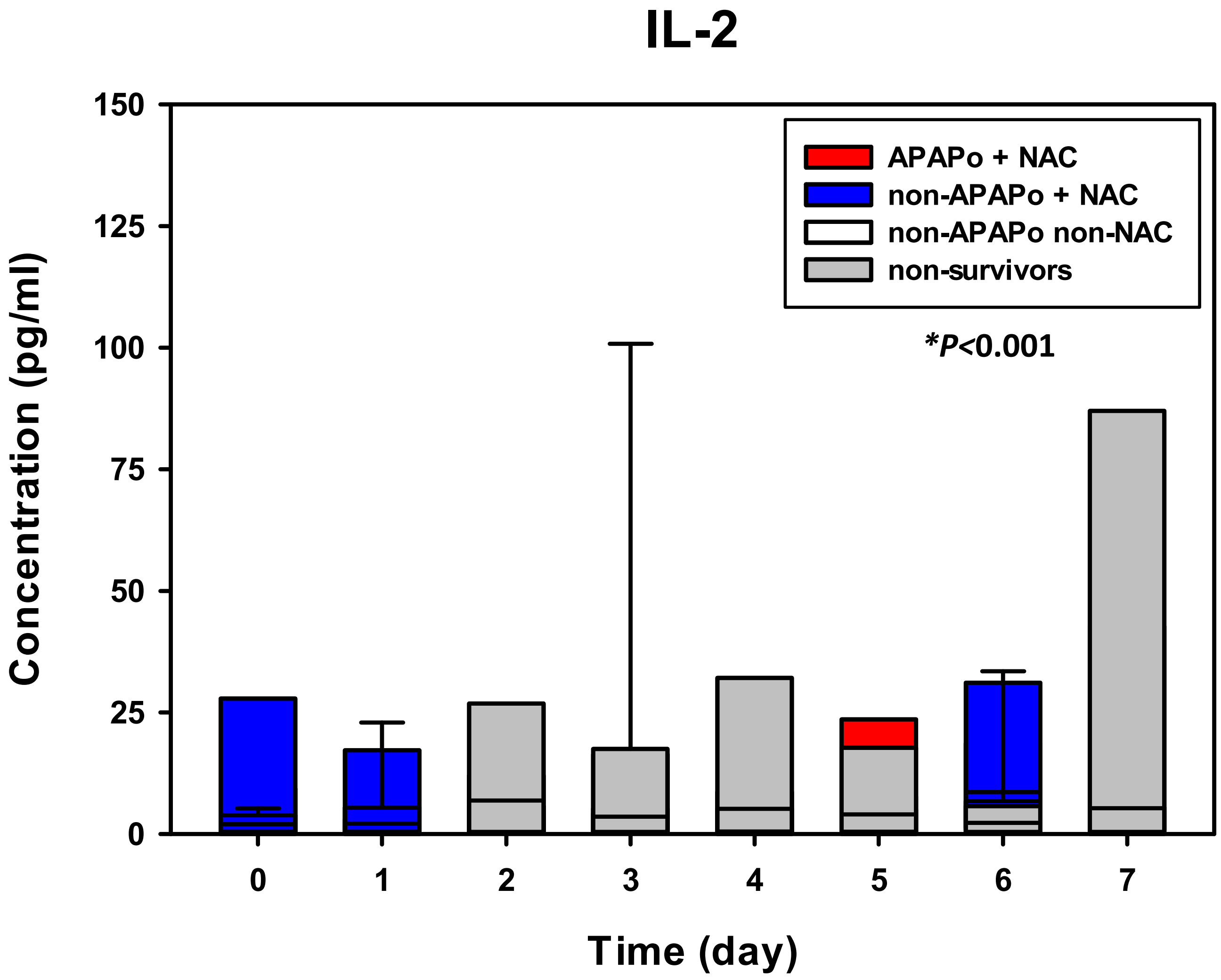

IL-2
150
125
100
75
50
25
0
Concentration (pg/ml)
APAPo + NAC
non-APAPo + NAC
non-APAPo non-NAC
non-survivors
*P<0.001
0
1
2
3
4
5
6
7
Time (day)

Suppl. Fig. 1

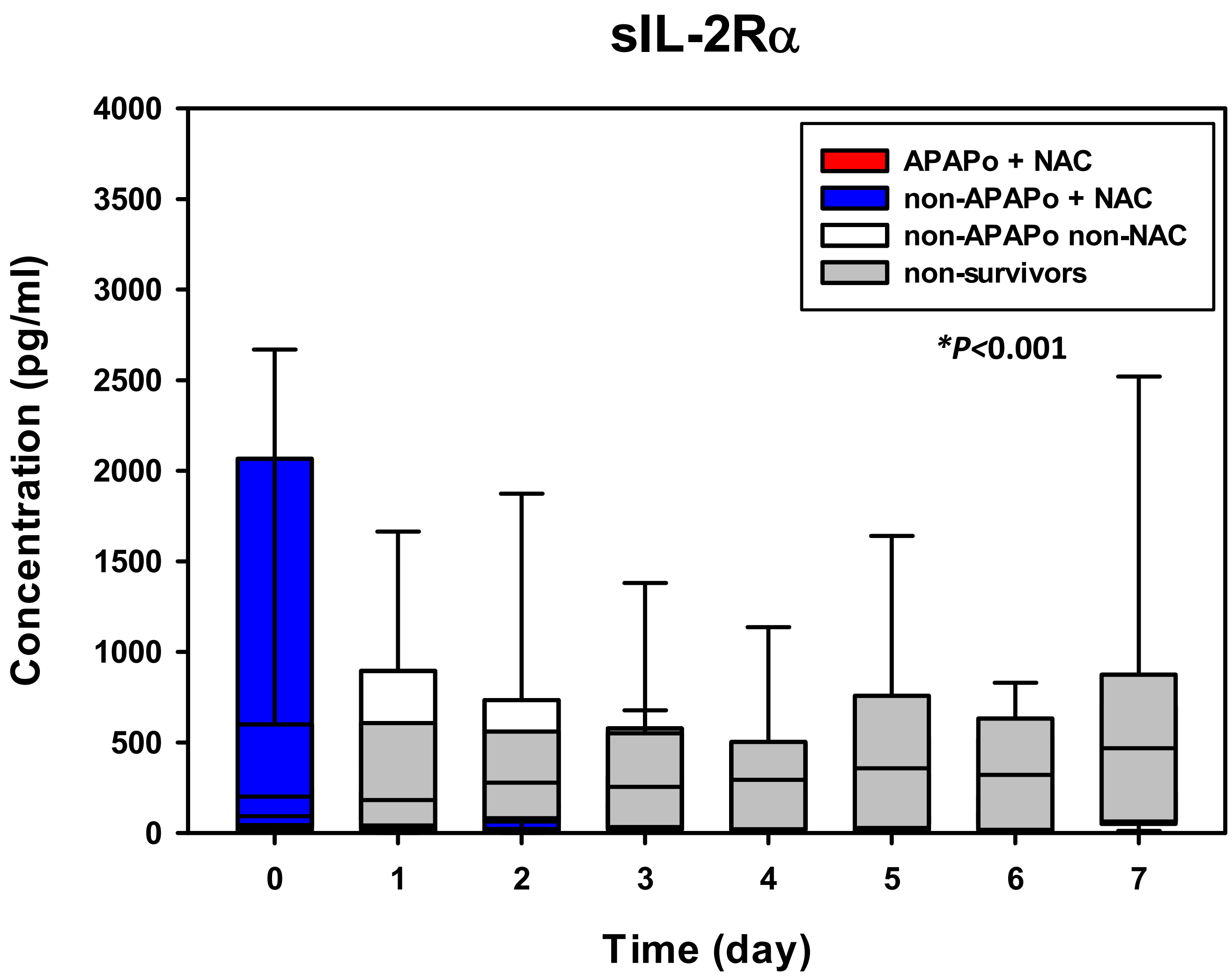
sIL-2Rα
4000
3500
3000
2500
2000
1500
1000
500
0
Concentration (pg/ml)
APAPo + NAC
non-APAPo + NAC
non-APAPo non-NAC
non-survivors
*P<0.001
0
1
2
3
4
5
6
7
Time (day)

Suppl. Fig. 1

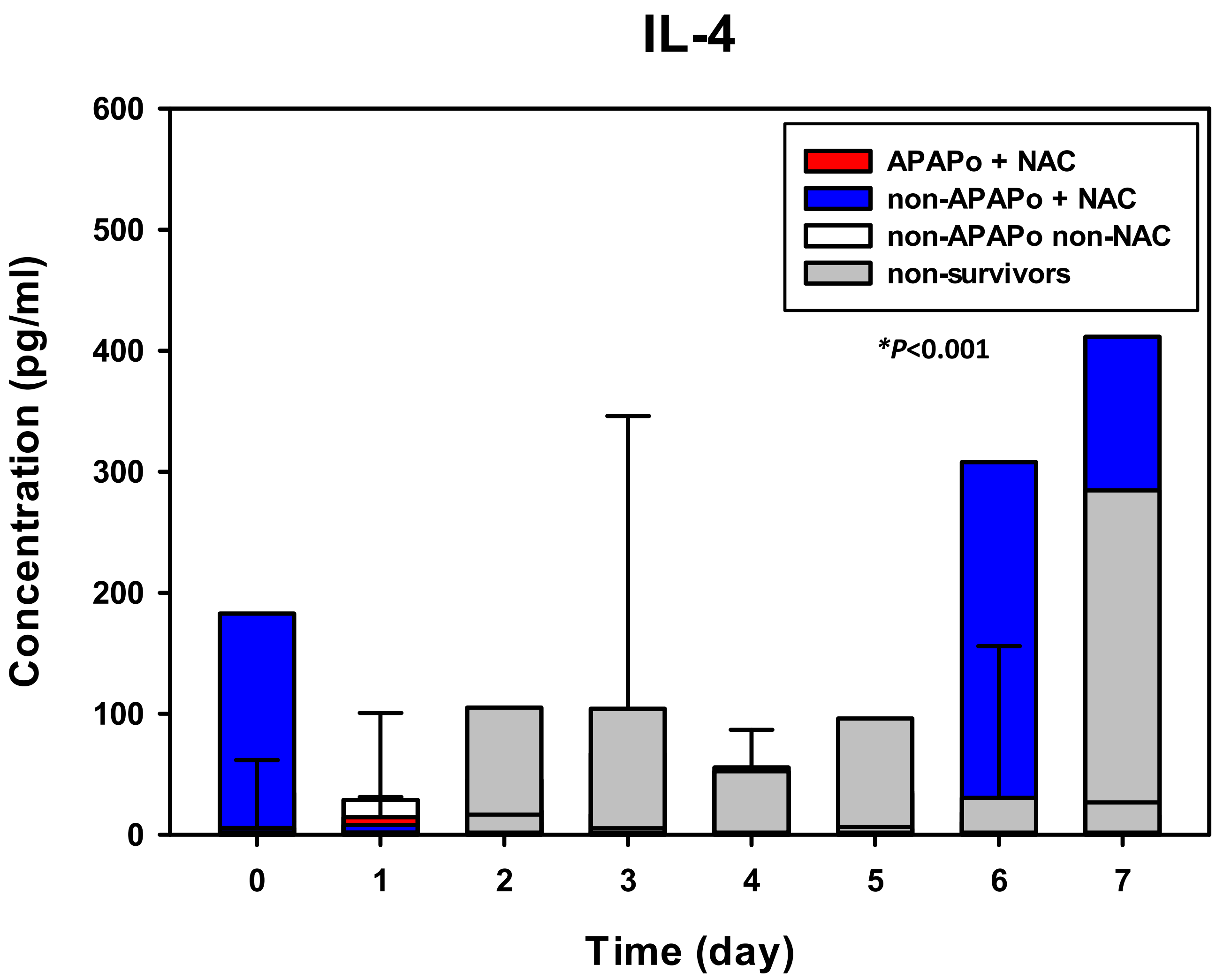
IL-4
APAPo + NAC
non-APAPo + NAC
non-APAPo non-NAC
non-survivors
*P<0.001
Concentration (pg/ml)
600
500
400
300
200
100
0
0
1
2
3
4
5
6
7
Time (day)

Suppl. Fig. 1

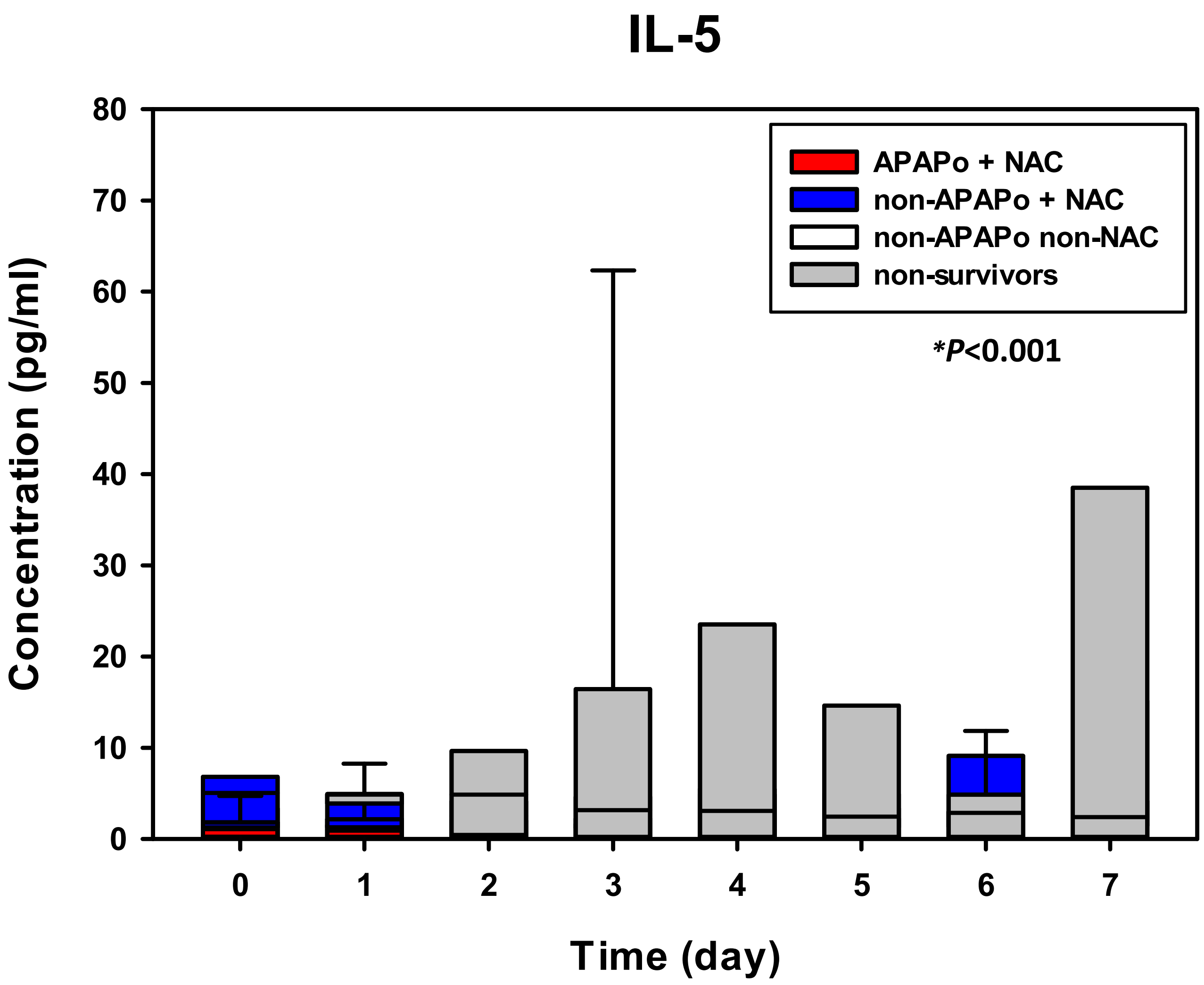

IL-5
80
70
60
50
40
30
20
10
0
Concentration (pg/ml)
APAPo + NAC
non-APAPo + NAC
non-APAPo non-NAC
non-survivors
*P<0.001
0
1
2
3
4
5
6
7
Time (day)

Suppl. Fig. 1

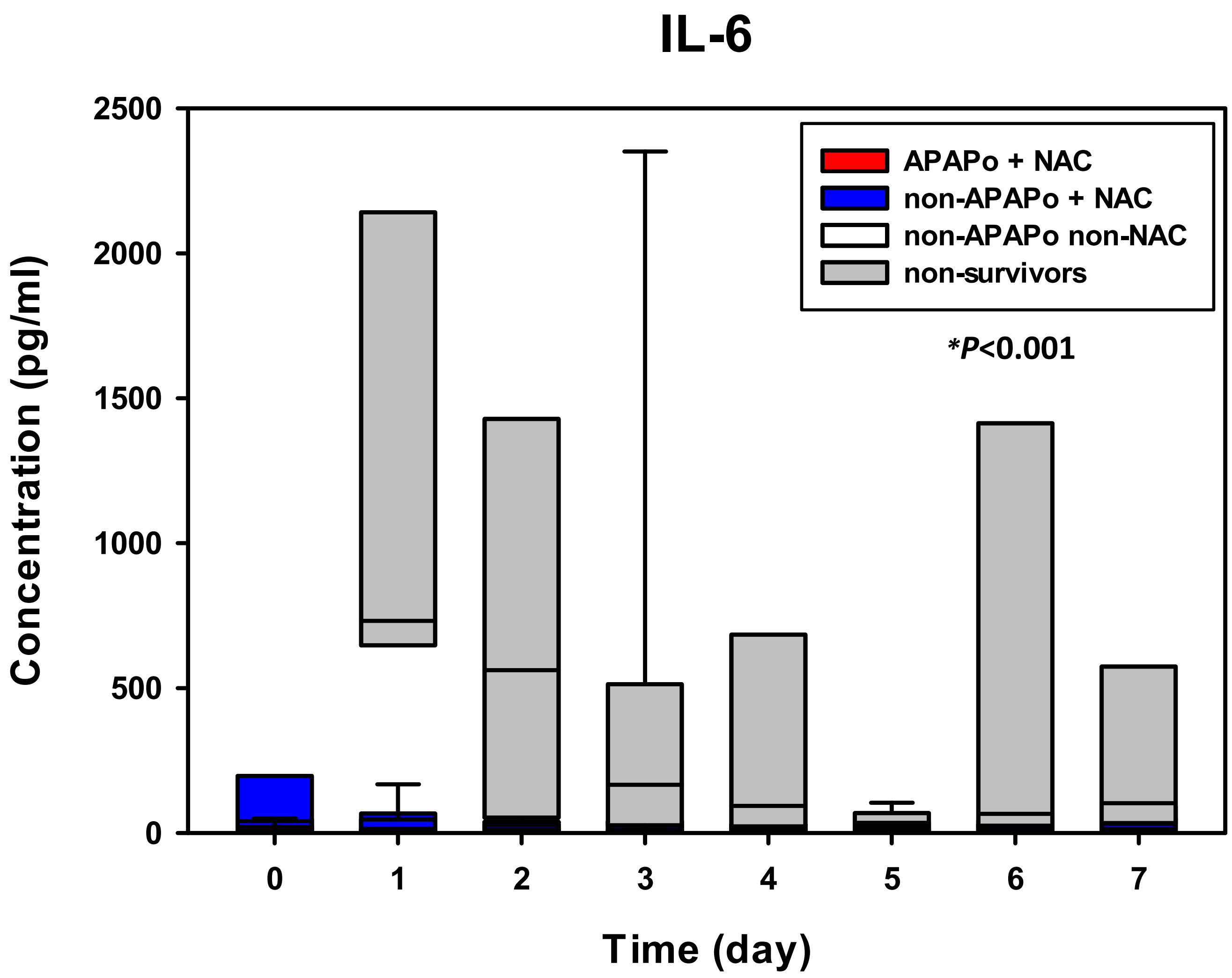

IL-6
2500
2000
1500
1000
500
0
Concentration (pg/ml)
APAPo + NAC
non-APAPo + NAC
non-APAPo non-NAC
non-survivors
*P<0.001
0
1
2
3
4
5
6
7
Time (day)

Suppl. Fig. 1

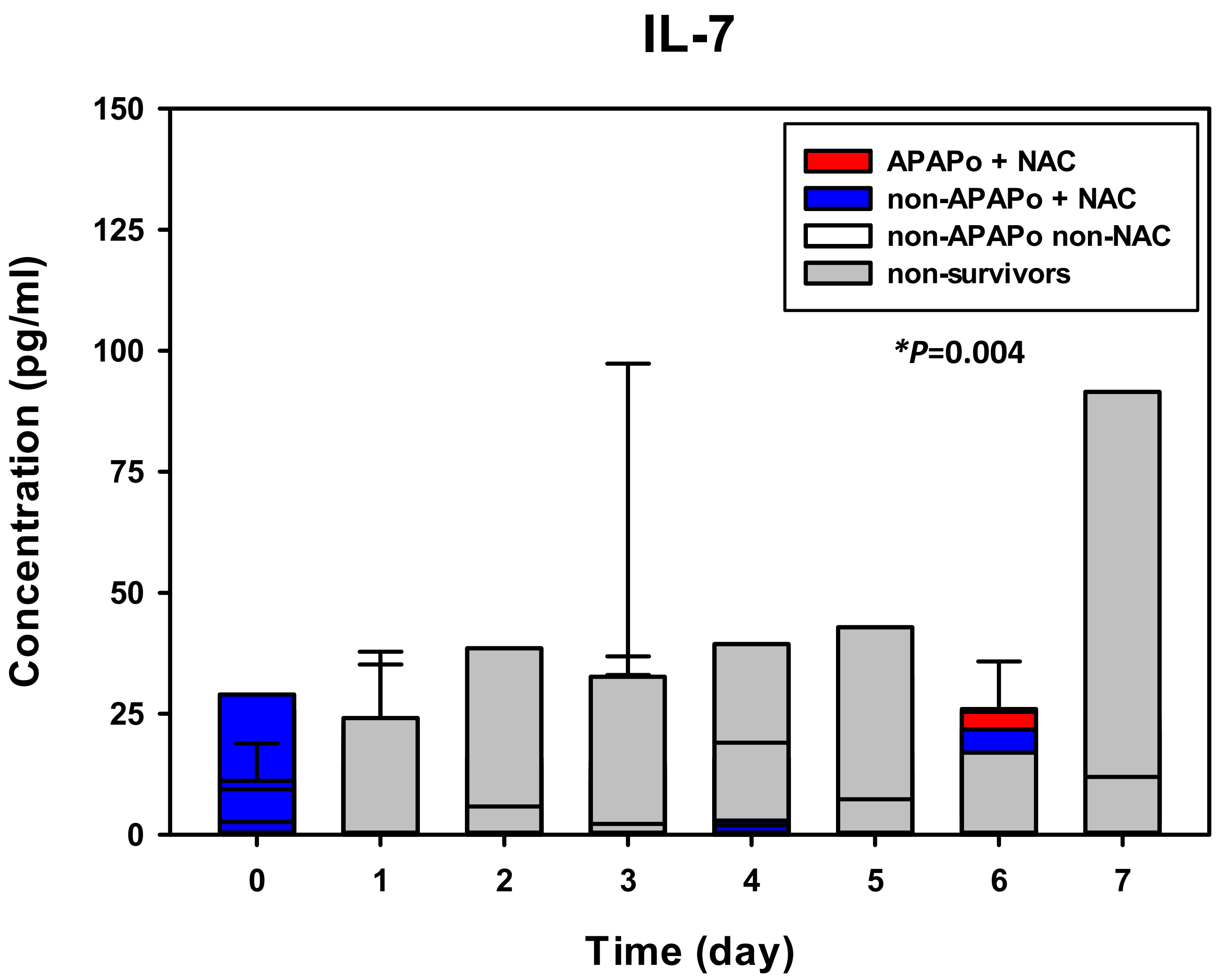

IL-7
Concentration (pg/ml)
150
125
100
75
50
25
0
APAPo + NAC
non-APAPo + NAC
non-APAPo non-NAC
non-survivors
*P=0.004
0
1
2
3
4
5
6
7
Time (day)

Suppl. Fig. 1

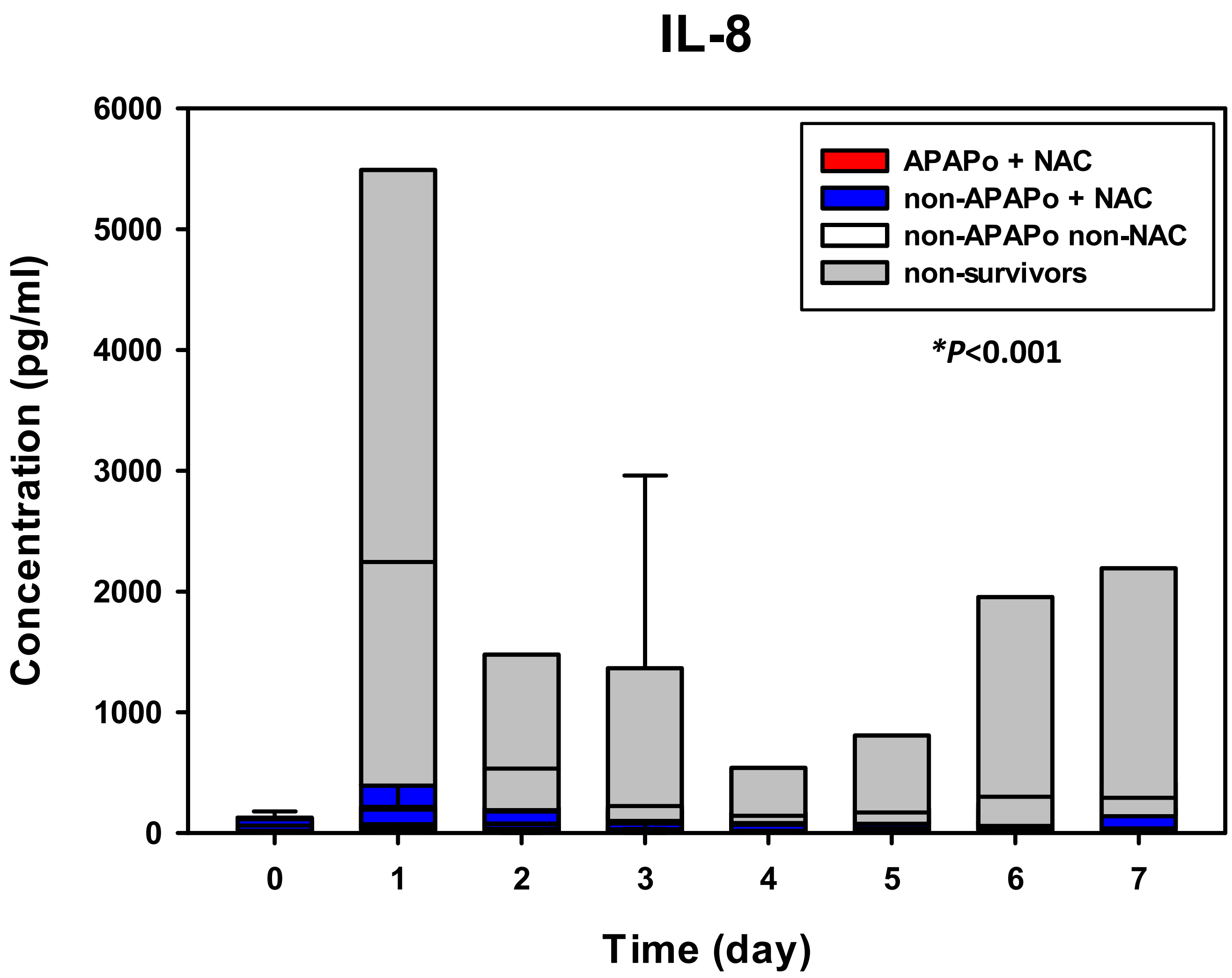
IL-8
6000
5000
4000
3000
2000
1000
0
Concentration (pg/ml)
APAPo + NAC
non-APAPo + NAC
non-APAPo non-NAC
non-survivors
*P<0.001
0
1
2
3
4
5
6
7
Time (day)

Suppl. Fig. 1

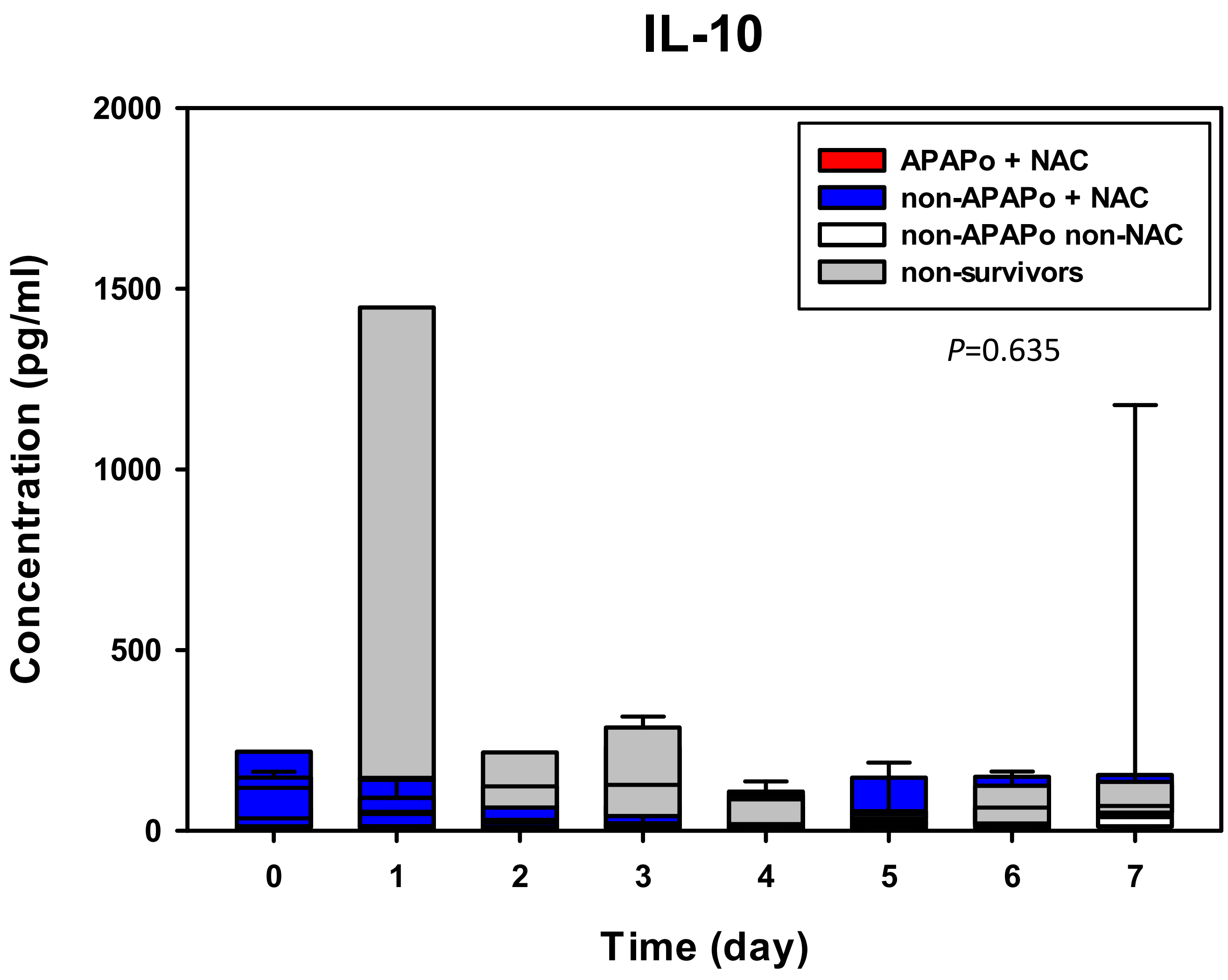
IL-10
APAPo + NAC
non-APAPo + NAC
non-APAPo non-NAC
non-survivors
P=0.635
Concentration (pg/ml)
2000
1500
1000
500
0
0
1
2
3
4
5
6
7
Time (day)

Suppl. Fig. 1

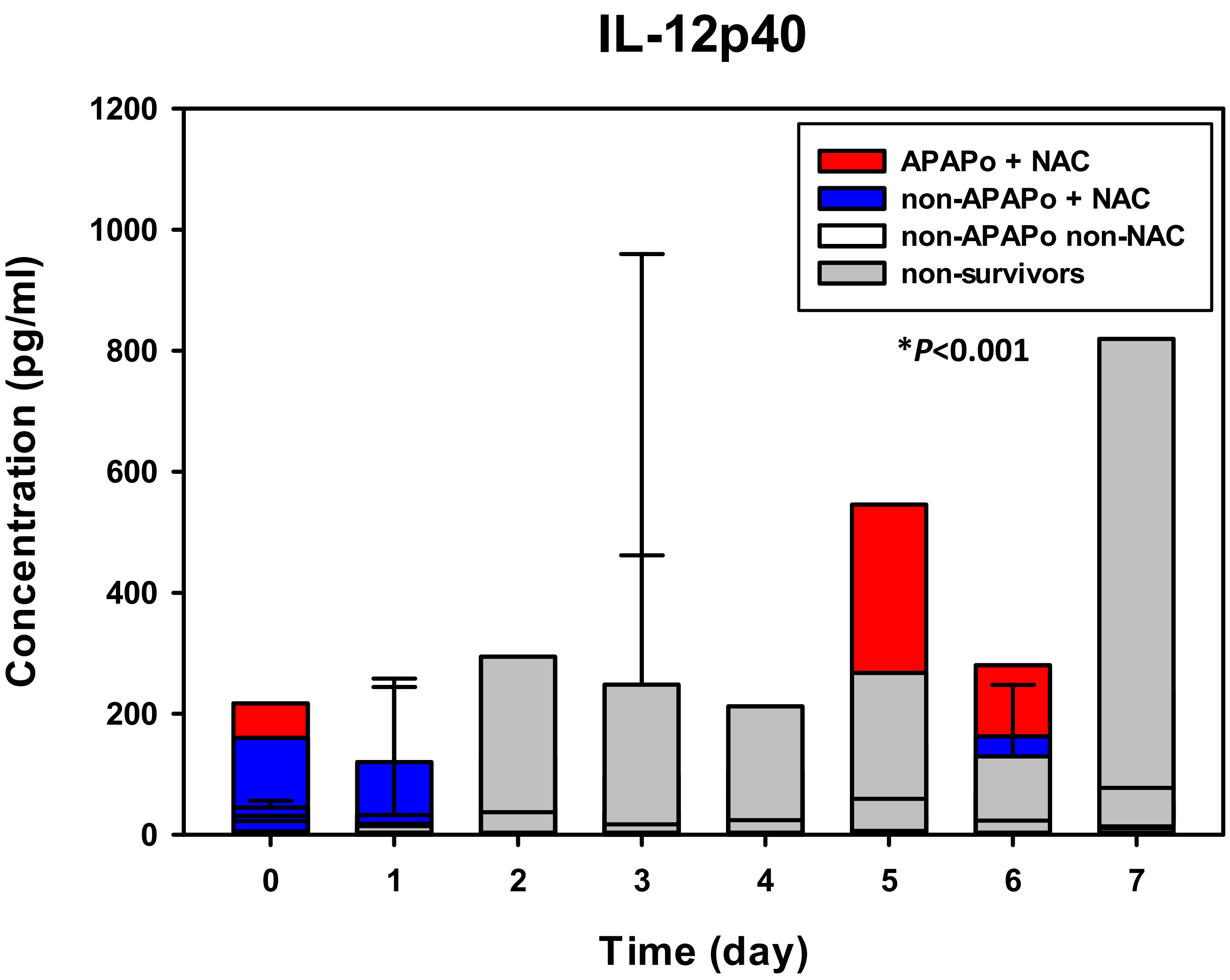

IL-12p40
1200
1000
800
600
400
200
0
Concentration (pg/ml)
APAPo + NAC
non-APAPo + NAC
non-APAPo non-NAC
non-survivors
*P<0.001
0
1
2
3
4
5
6
7
Time (day)

Suppl. Fig. 1

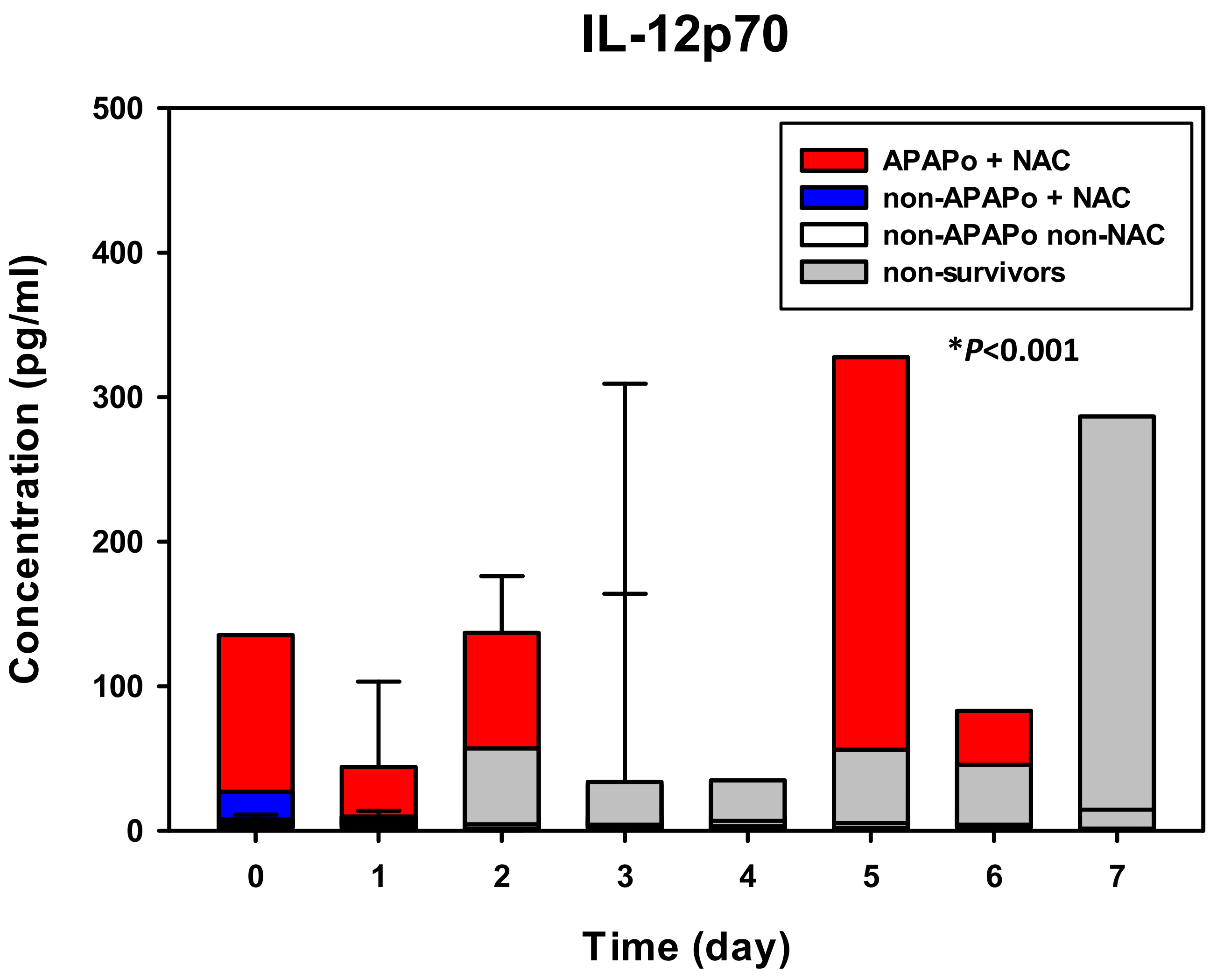
IL-12p70
500
400
300
200
100
0
Concentration (pg/ml)
APAPo + NAC
non-APAPo + NAC
non-APAPo non-NAC
non-survivors
*P<0.001
0
1
2
3
4
5
6
7
Time (day)

Suppl. Fig. 1

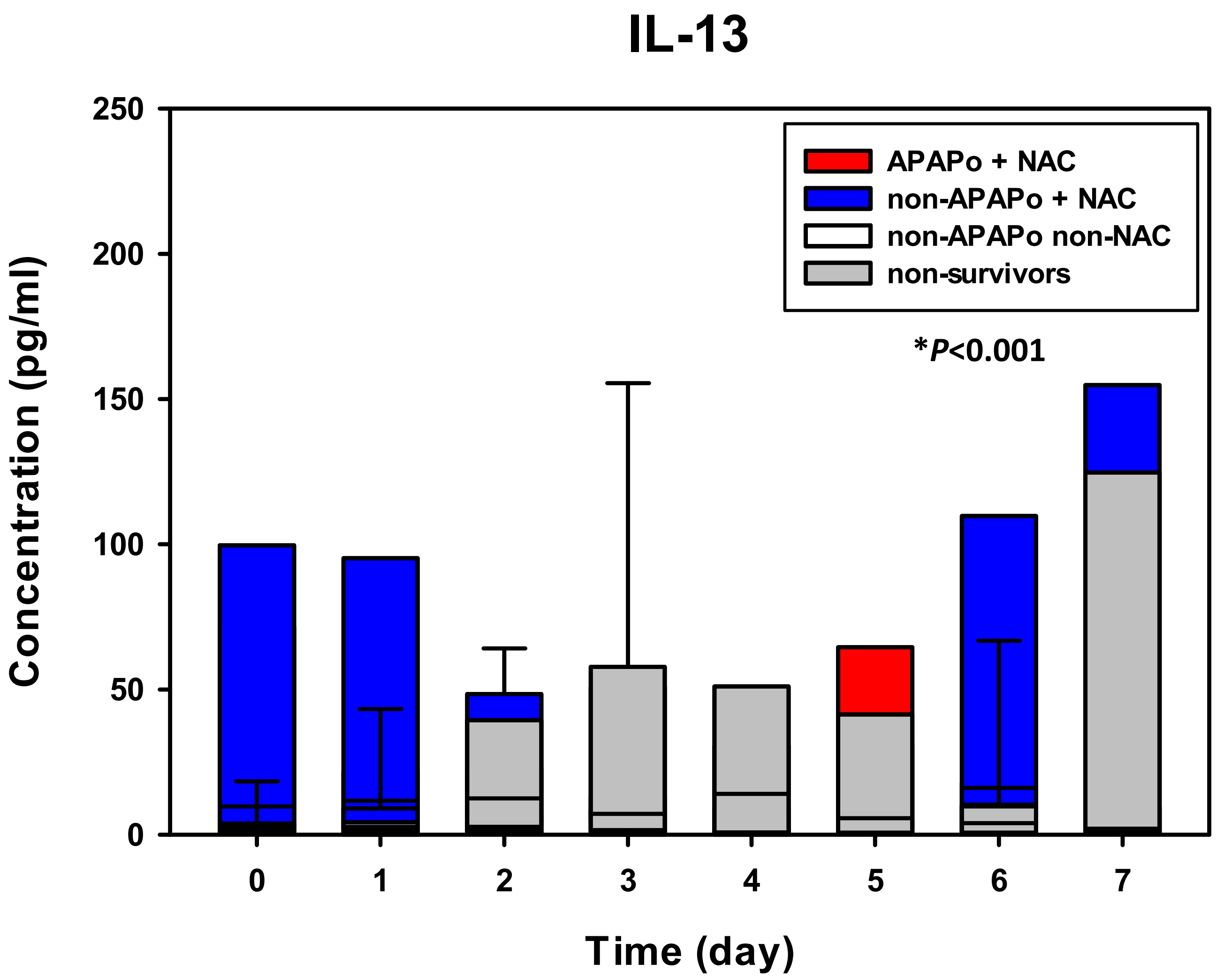

IL-13
Concentration (pg/ml)
0
50
100
150
200
250
0
1
2
3
4
5
6
7
Time (day)
APAPo + NAC
non-APAPo + NAC
non-APAPo non-NAC
non-survivors
*P<0.001

Suppl. Fig. 1

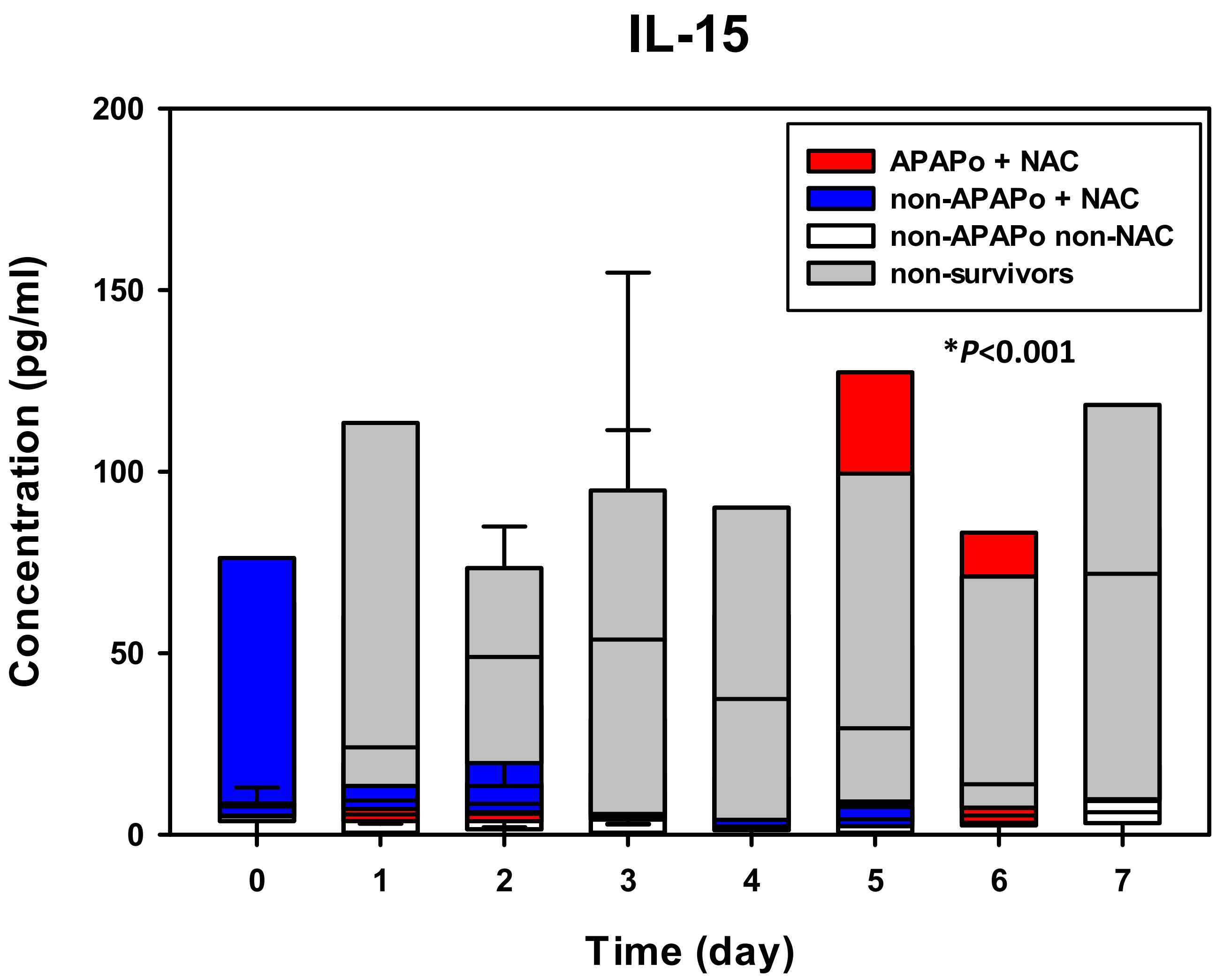
IL-15
APAPo + NAC
non-APAPo + NAC
non-APAPo non-NAC
non-survivors
*P<0.001
Concentration (pg/ml)
200
150
100
50
0
0
1
2
3
4
5
6
7
Time (day)

Suppl. Fig. 1

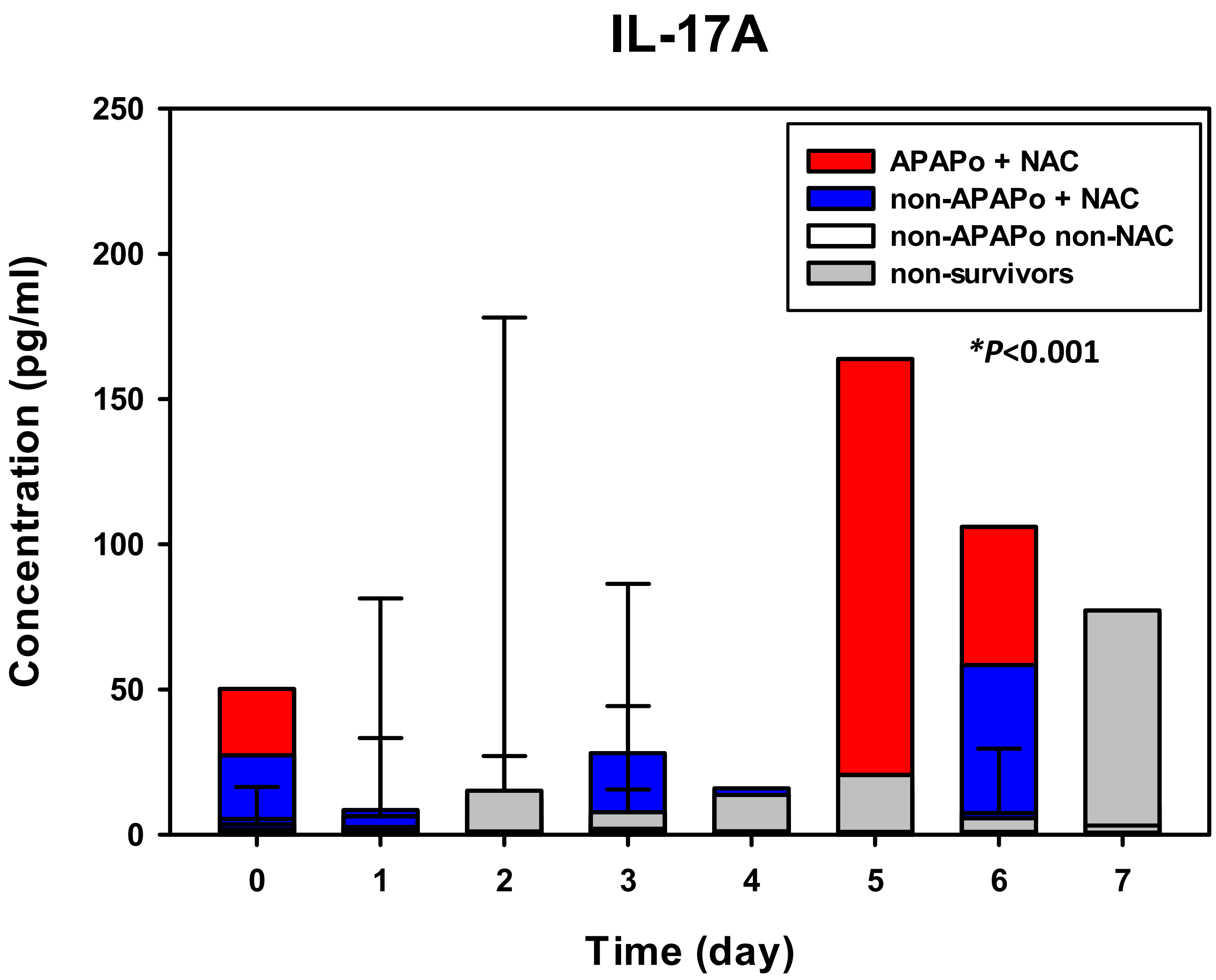
IL-17A
APAPo + NAC
non-APAPo + NAC
non-APAPo non-NAC
non-survivors
*P<0.001
Concentration (pg/ml)
0
50
100
150
200
250
Time (day)
0
1
2
3
4
5
6
7

Suppl. Fig. 1

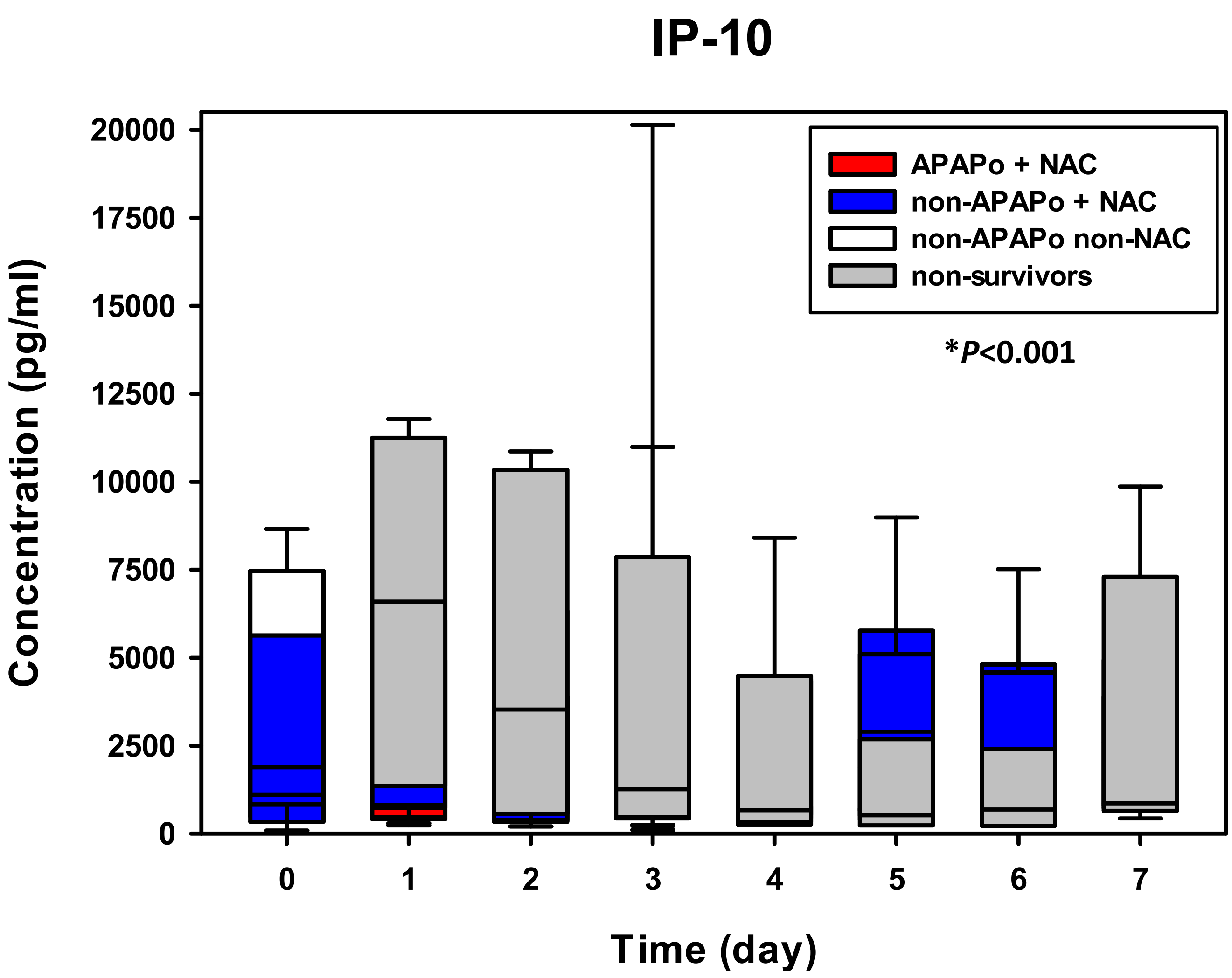

IP-10
Concentration (pg/ml)
20000
17500
15000
12500
10000
7500
5000
2500
0
APAPo + NAC
non-APAPo + NAC
non-APAPo non-NAC
non-survivors
*P<0.001
0
1
2
3
4
5
6
7
Time (day)

Suppl. Fig. 1

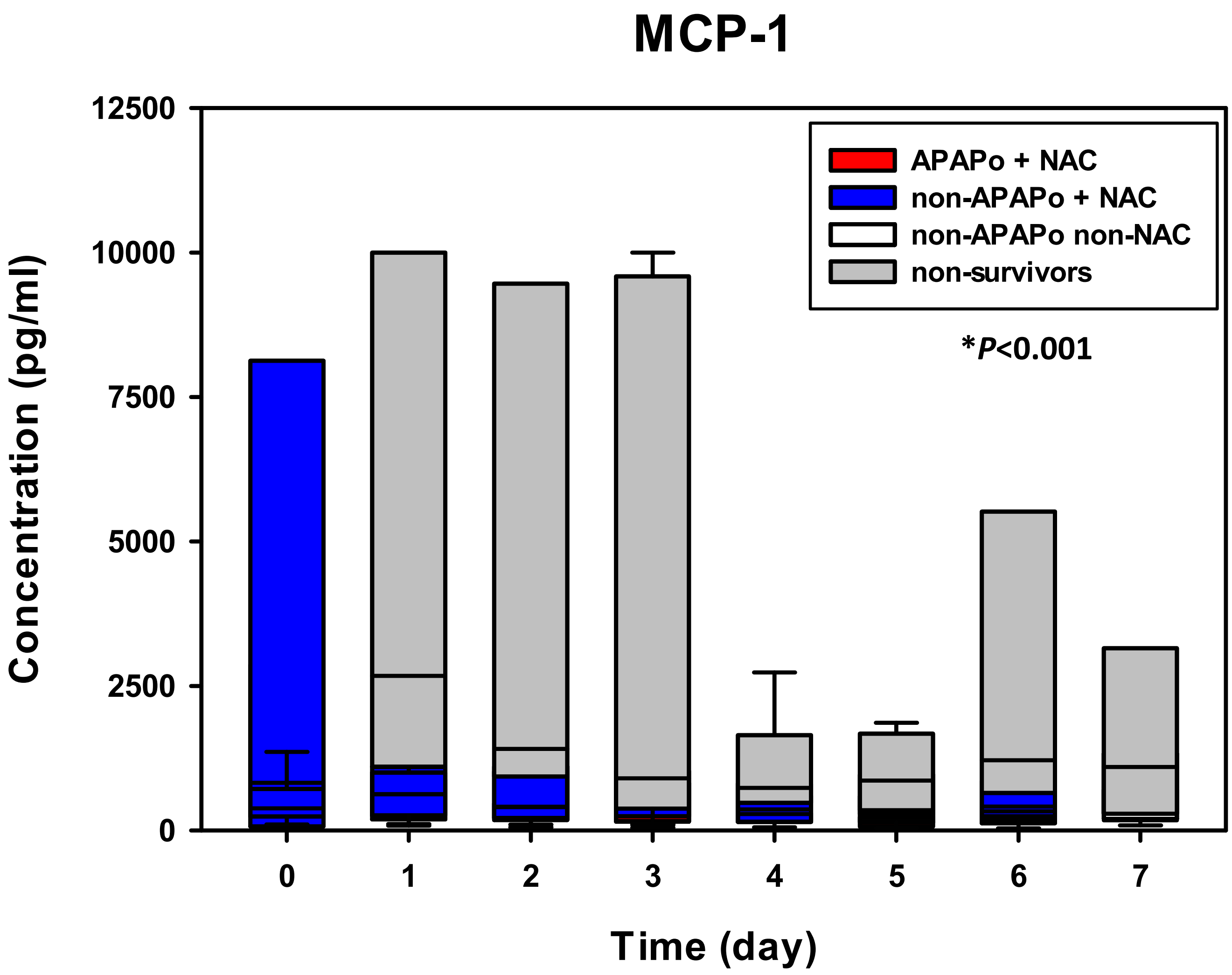

MCP-1
Concentration (pg/ml)
12500
10000
7500
5000
2500
0
0
1
2
3
4
5
6
7
Time (day)
APAPo + NAC
non-APAPo + NAC
non-APAPo non-NAC
non-survivors
*P<0.001

Suppl. Fig. 1

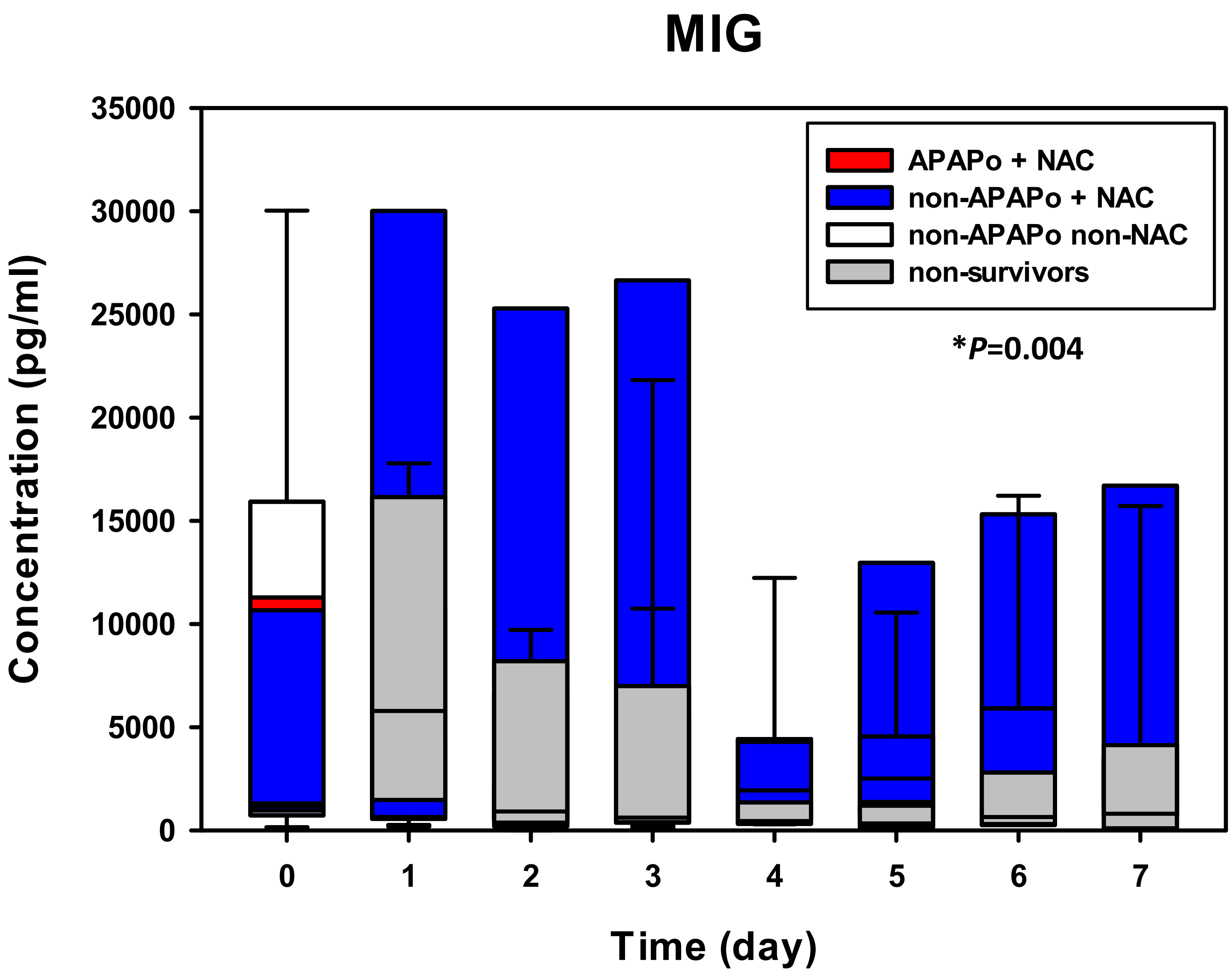
MIG
35000
30000
25000
20000
15000
10000
5000
0
Concentration (pg/ml)
APAPo + NAC
non-APAPo + NAC
non-APAPo non-NAC
non-survivors
*P=0.004
0
1
2
3
4
5
6
7
Time (day)

Suppl. Fig. 1

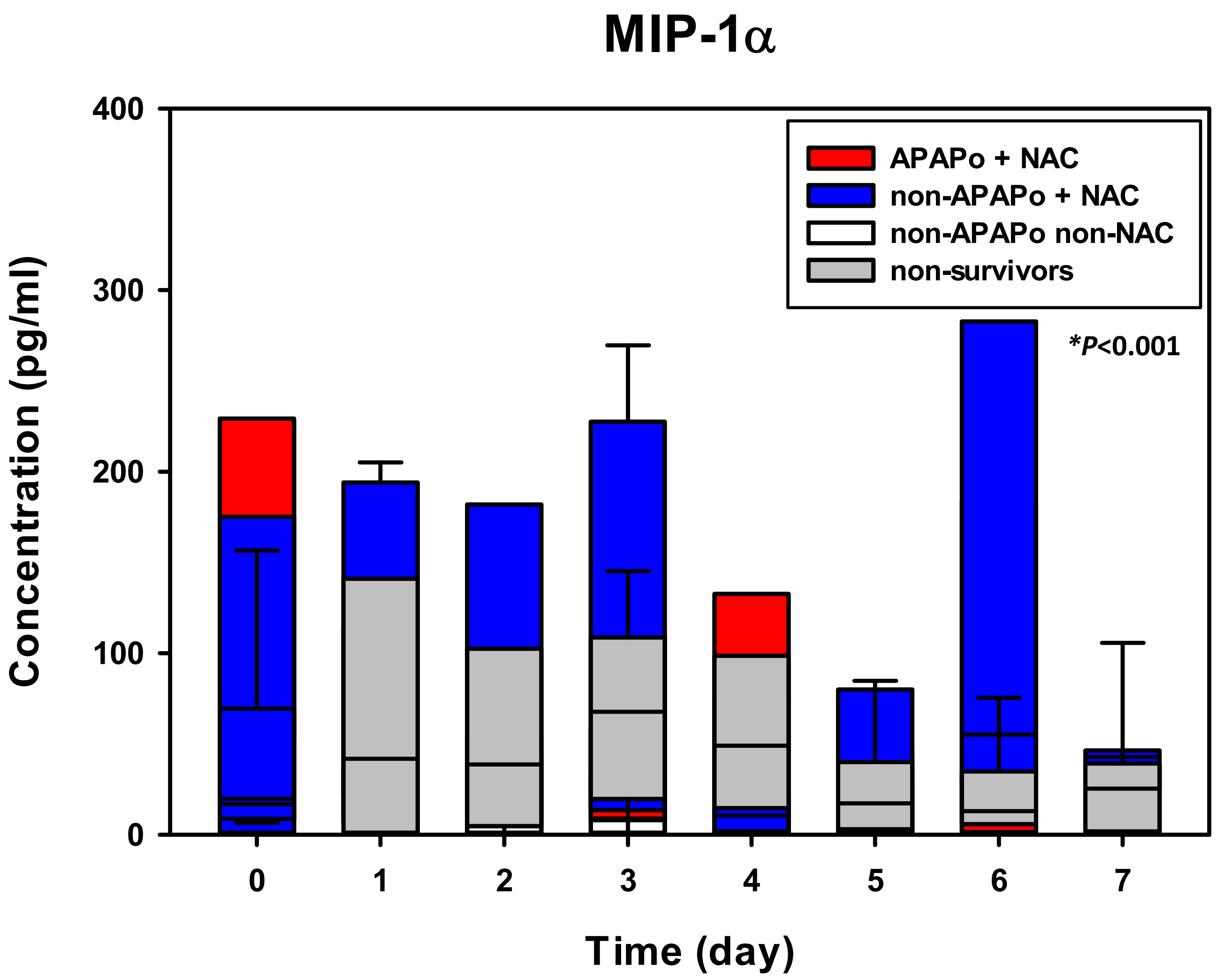
MIP-1α
400
300
200
100
0
Concentration (pg/ml)
APAPo + NAC
non-APAPo + NAC
non-APAPo non-NAC
non-survivors
*P<0.001
0
1
2
3
4
5
6
7
Time (day)

Suppl. Fig. 1

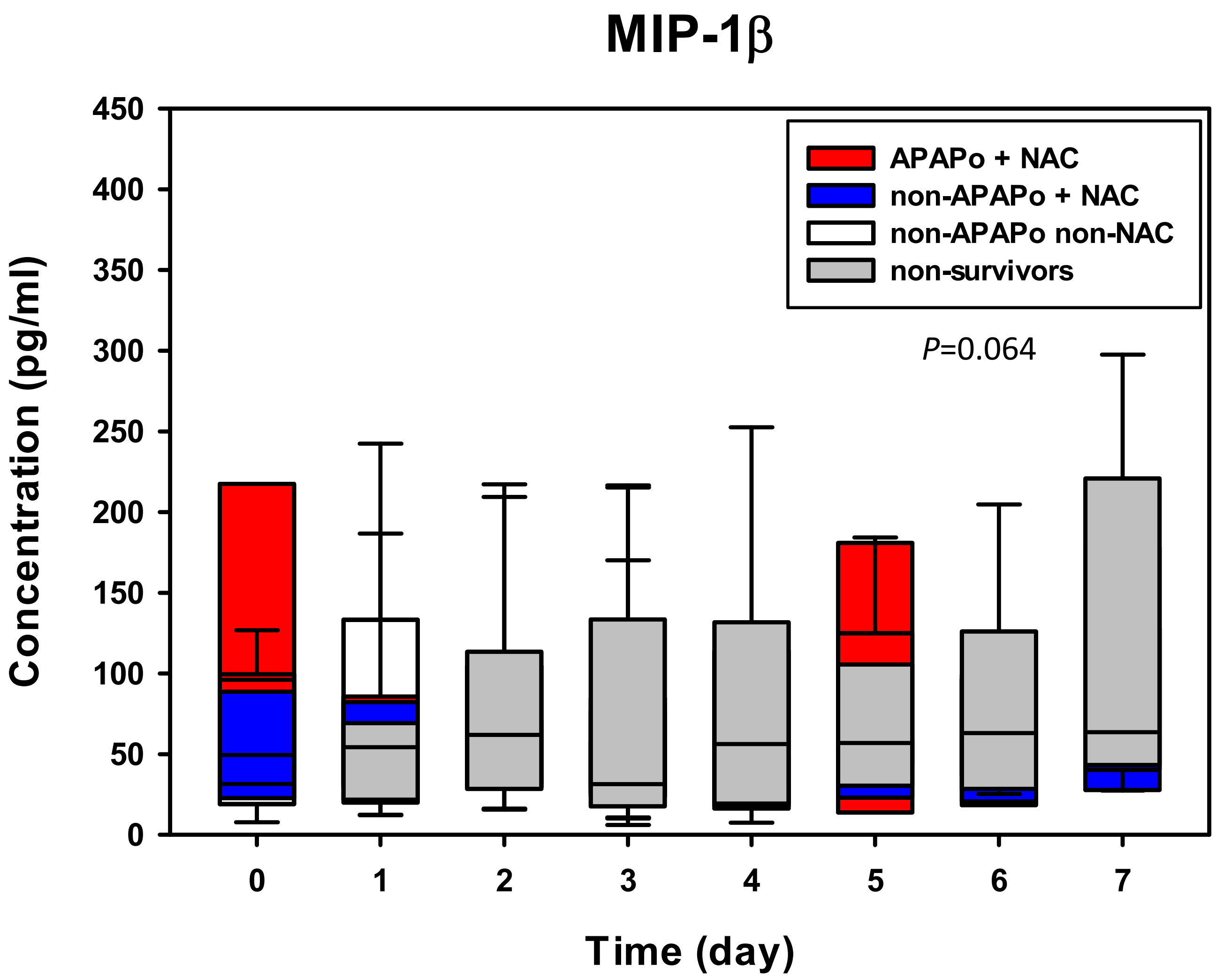

MIP-1β
APAPo + NAC
non-APAPo + NAC
non-APAPo non-NAC
non-survivors
P=0.064
Concentration (pg/ml)
450
400
350
300
250
200
150
100
50
0
0
1
2
3
4
5
6
7
Time (day)

Suppl. Fig. 1

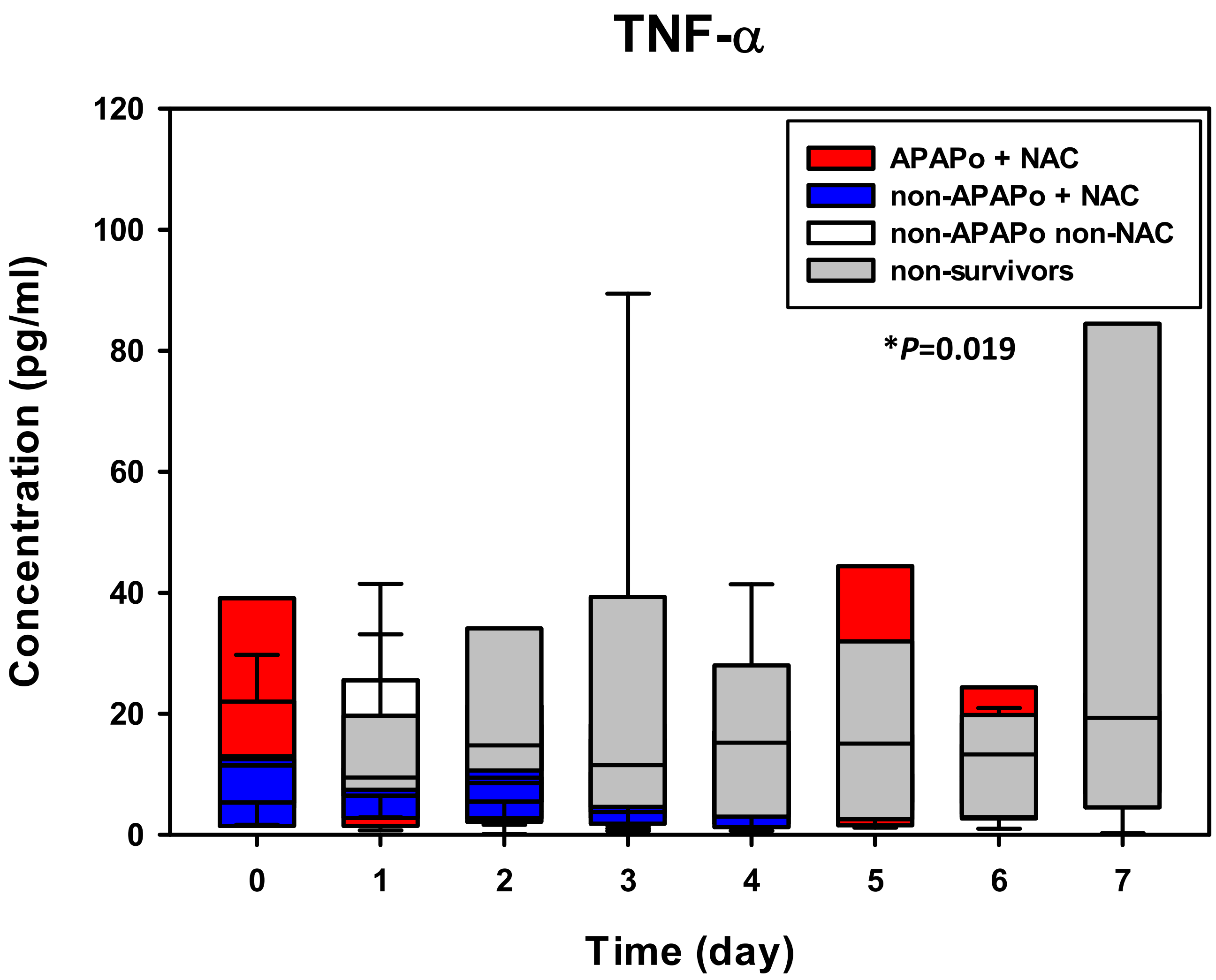
TNF-α
120
100
80
60
40
20
0
Concentration (pg/ml)
APAPo + NAC
non-APAPo + NAC
non-APAPo non-NAC
non-survivors
*P=0.019
0
1
2
3
4
5
6
7
Time (day)

Suppl. Fig. 1

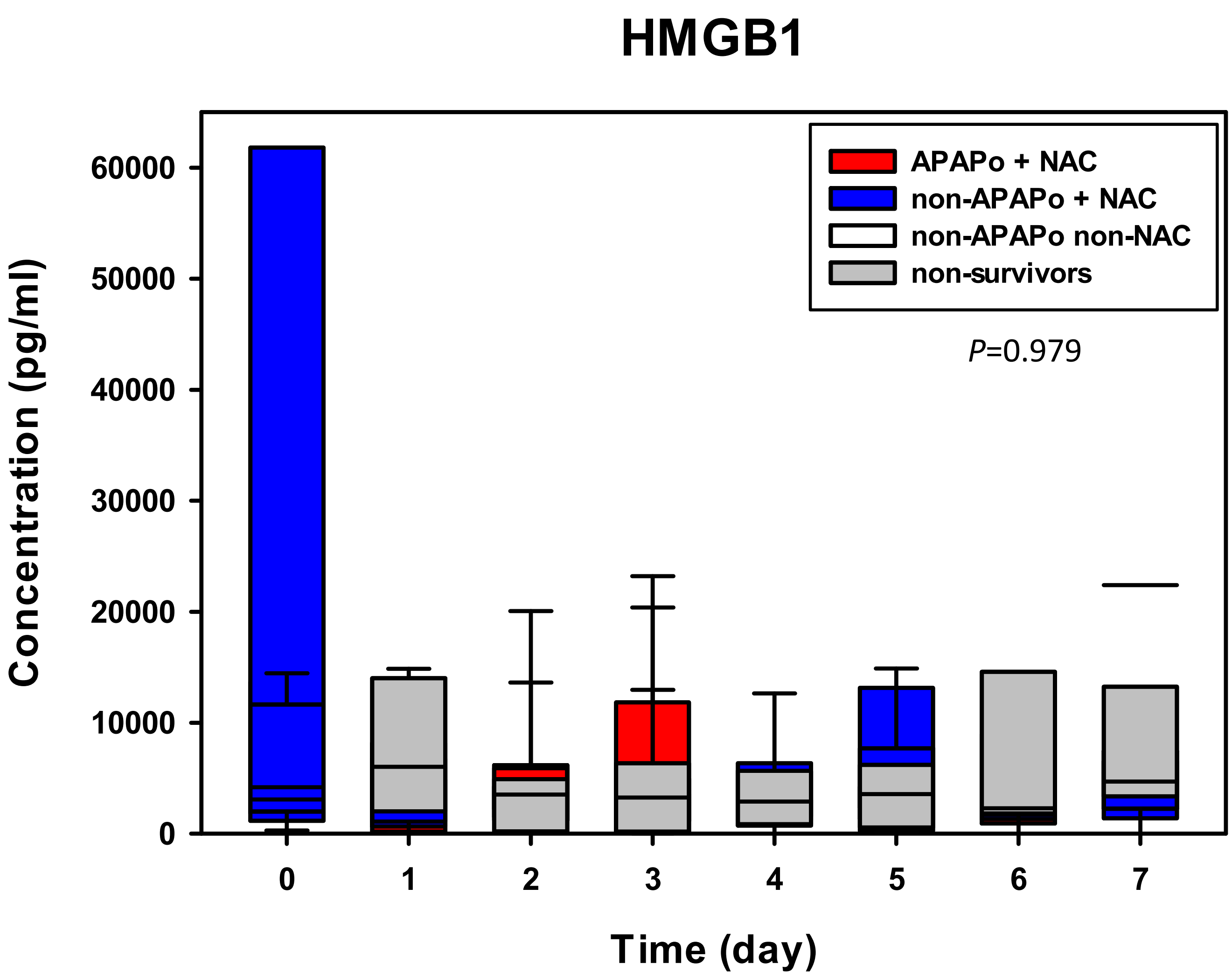
HMGB1
APAPo + NAC
non-APAPo + NAC
non-APAPo non-NAC
non-survivors
P=0.979
Concentration (pg/ml)
60000
50000
40000
30000
20000
10000
0
0
1
2
3
4
5
6
7
Time (day)

Suppl. Fig. 1

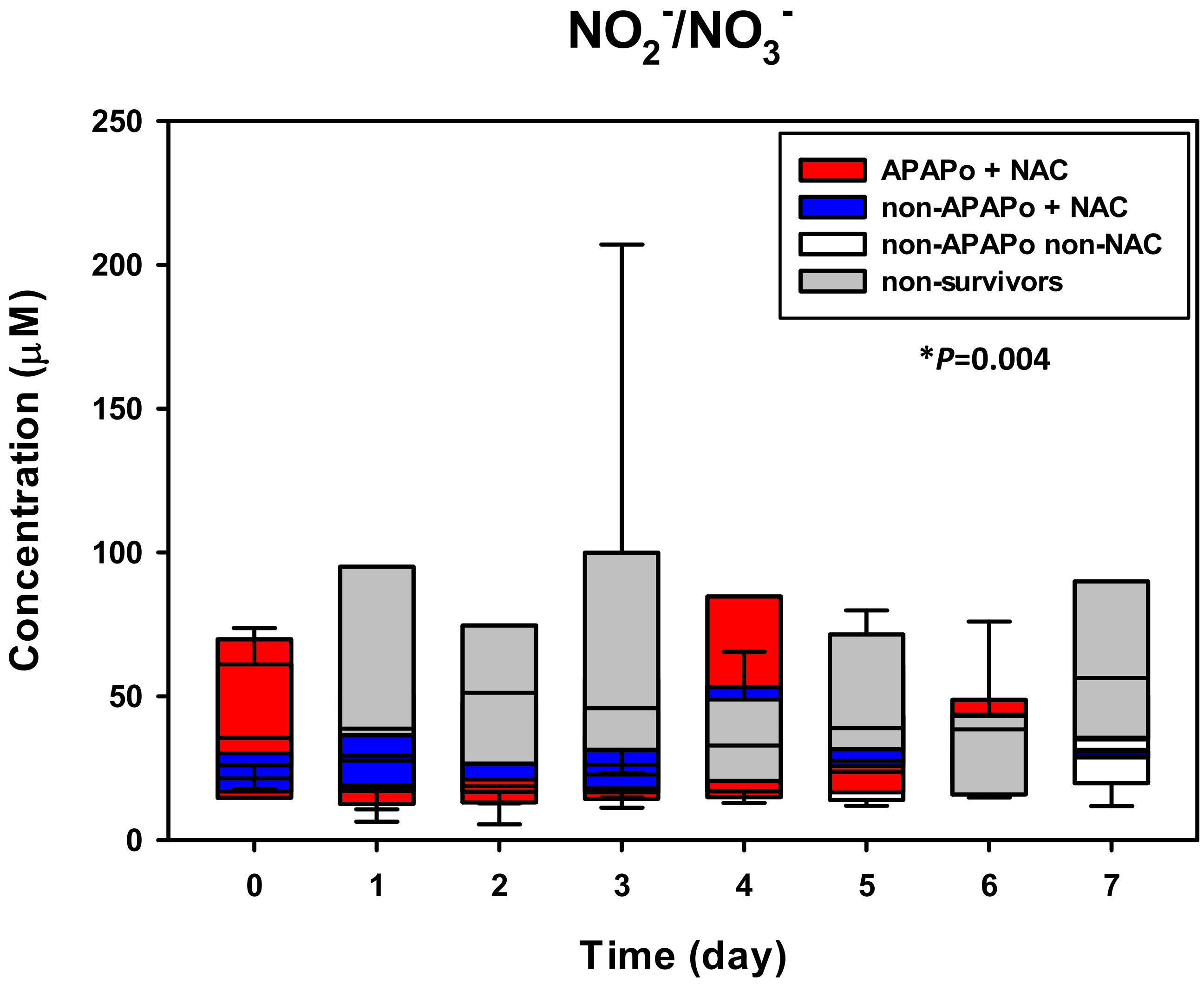
NO2-/NO3-
APAPo + NAC
non-APAPo + NAC
non-APAPo non-NAC
non-survivors
*P=0.004
Concentration (μM)
250
200
150
100
50
0
0
1
2
3
4
5
6
7
Time (day)

Suppl. Fig. 2

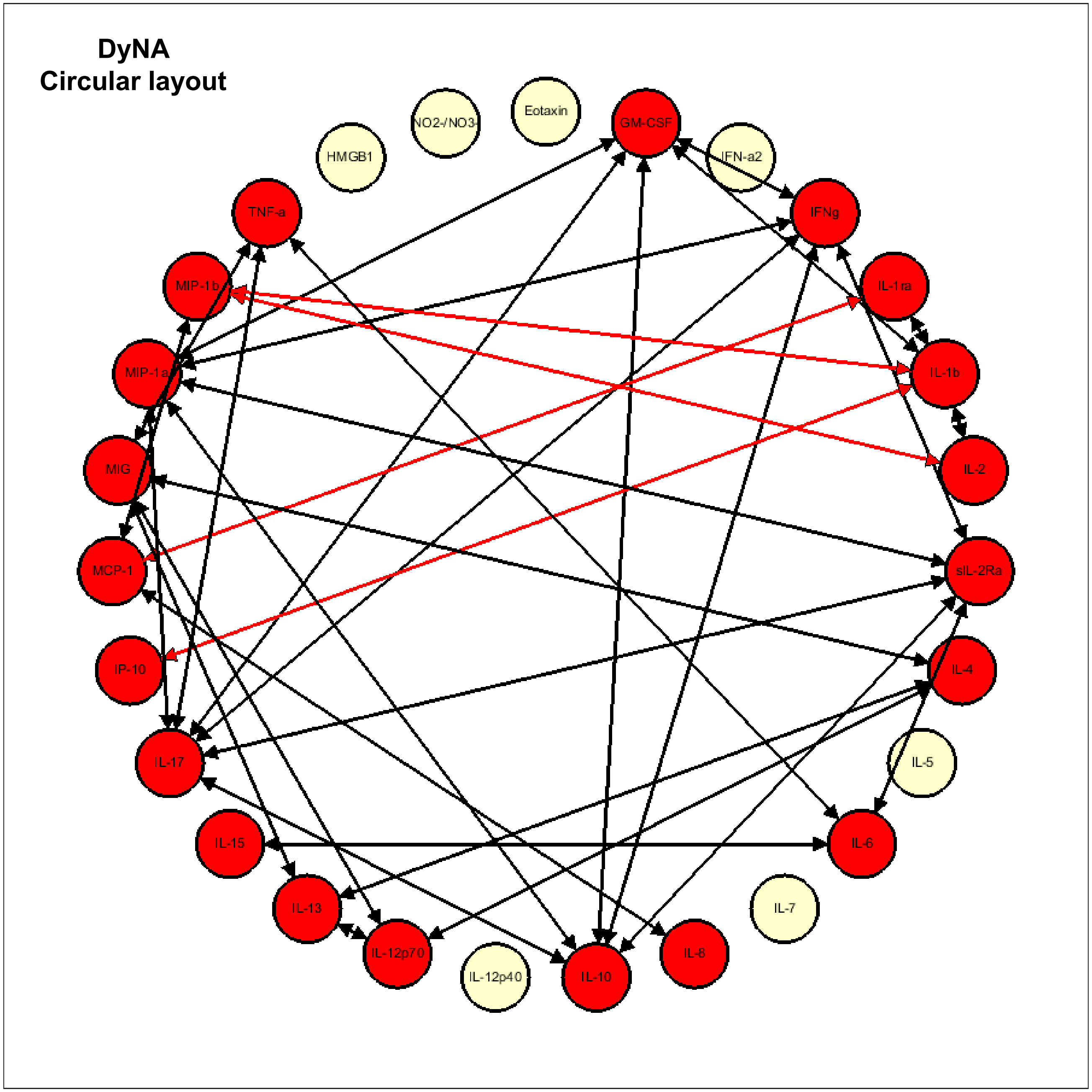
DyNA
Circular layout
HMGB1
NO2-/NO3-
Eotaxin
GM-CSF
IFN-a2
IFNg
IL-1ra
IL-1b
IL-2
sIL-2Ra
IL-4
IL-5
IL-6
IL-7
IL-8
IL-10
IL-12p40
IL-12p70
IL-13
IL-15
IL-17
IP-10
MCP-1
MIG
MIP-1a
MIP-1b
TNF-a

Suppl. Fig. 3

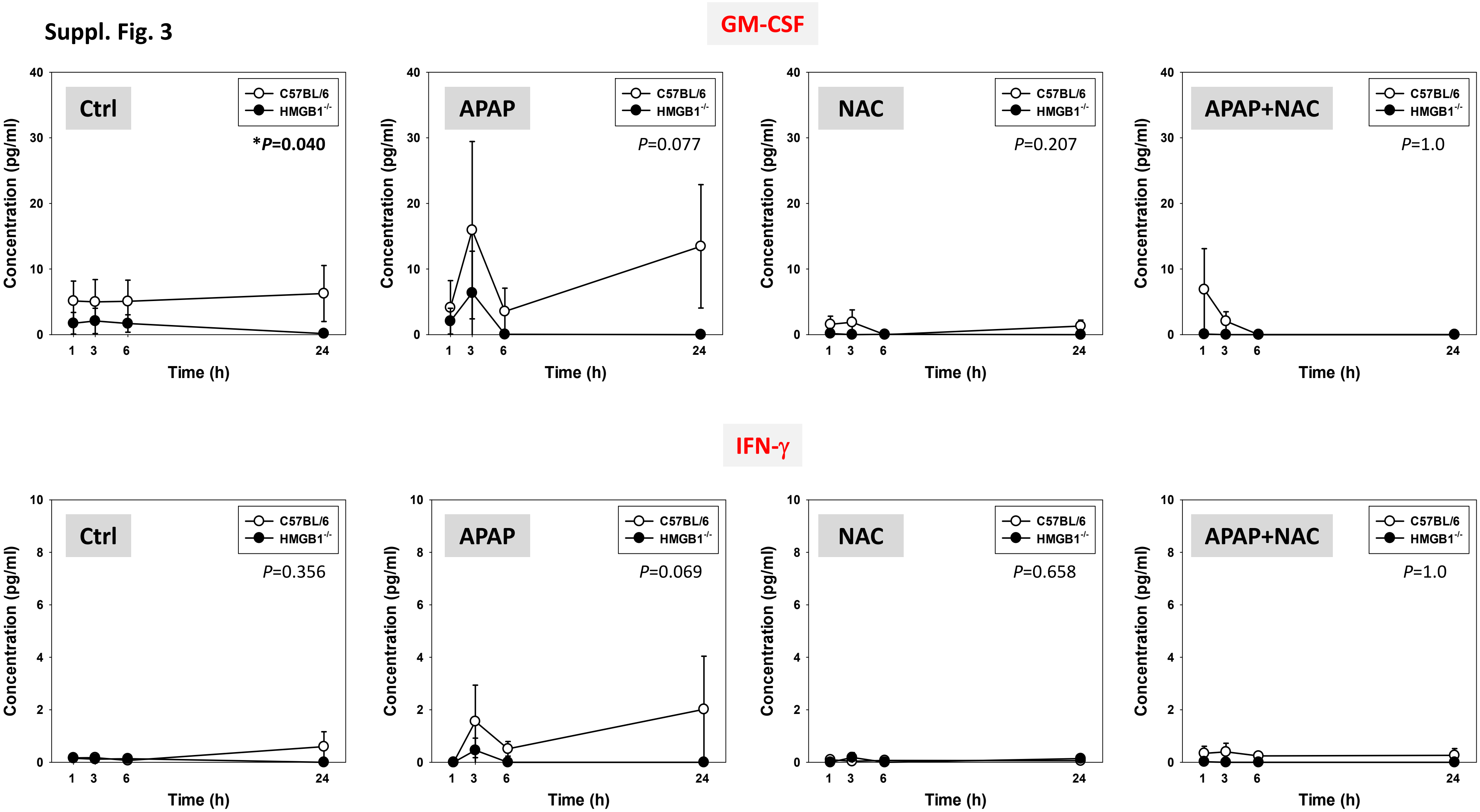
GM-CSF
Ctrl
C57BL/6
HMGB1-/-
*P=0.040
APAP
P=0.077
NAC
P=0.207
APAP+NAC
P=1.0
Concentration (pg/ml)
Time (h)
IFN-γ
Ctrl
P=0.356
APAP
P=0.069
NAC
P=0.658
APAP+NAC
P=1.0

Suppl. Fig. 3

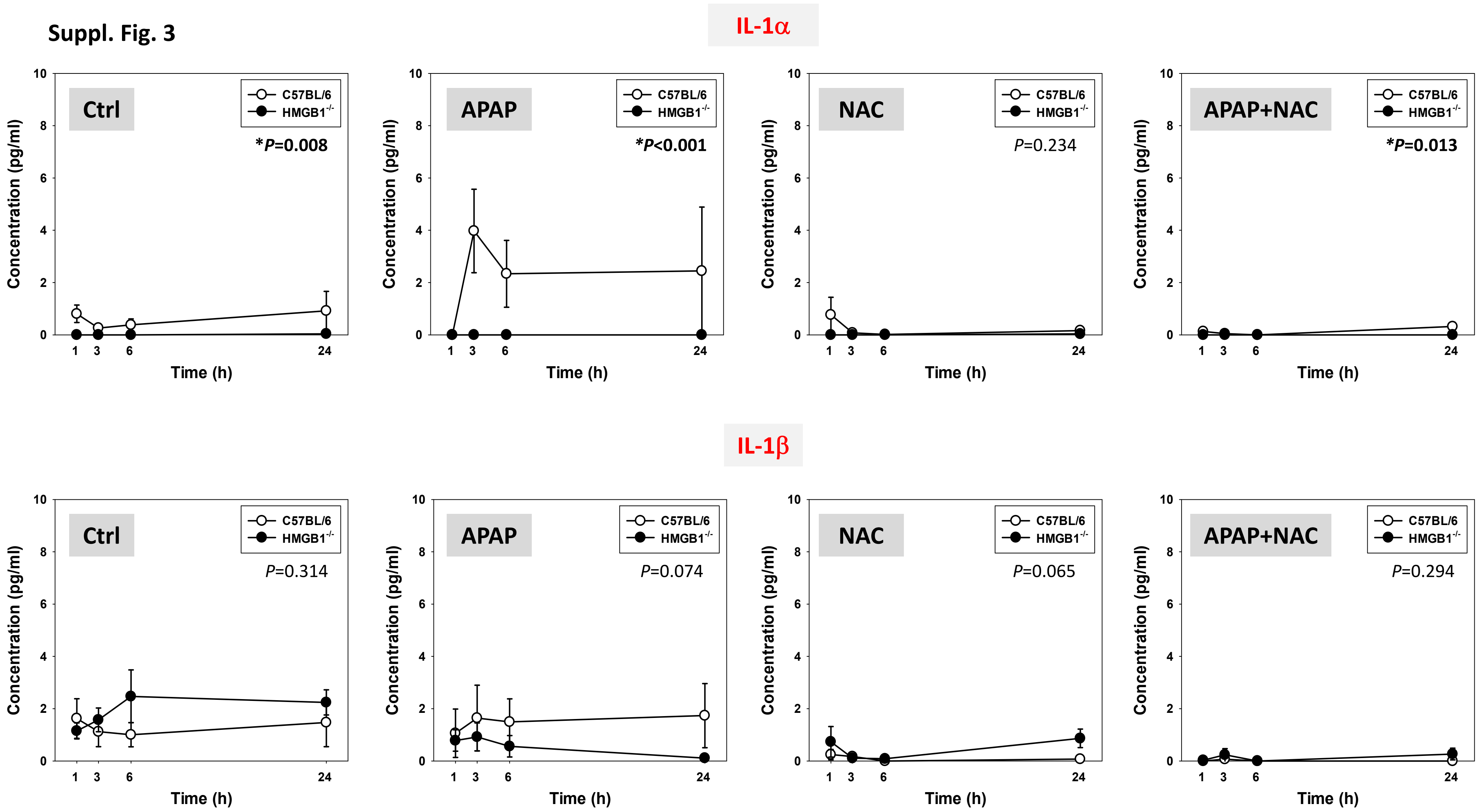
IL-1α
Ctrl
APAP
NAC
APAP+NAC
C57BL/6
HMGB1-/-
*P=0.008
*P<0.001
P=0.234
*P=0.013
Concentration (pg/ml)
Time (h)
IL-1β
P=0.314
P=0.074
P=0.065
P=0.294

Suppl. Fig. 3

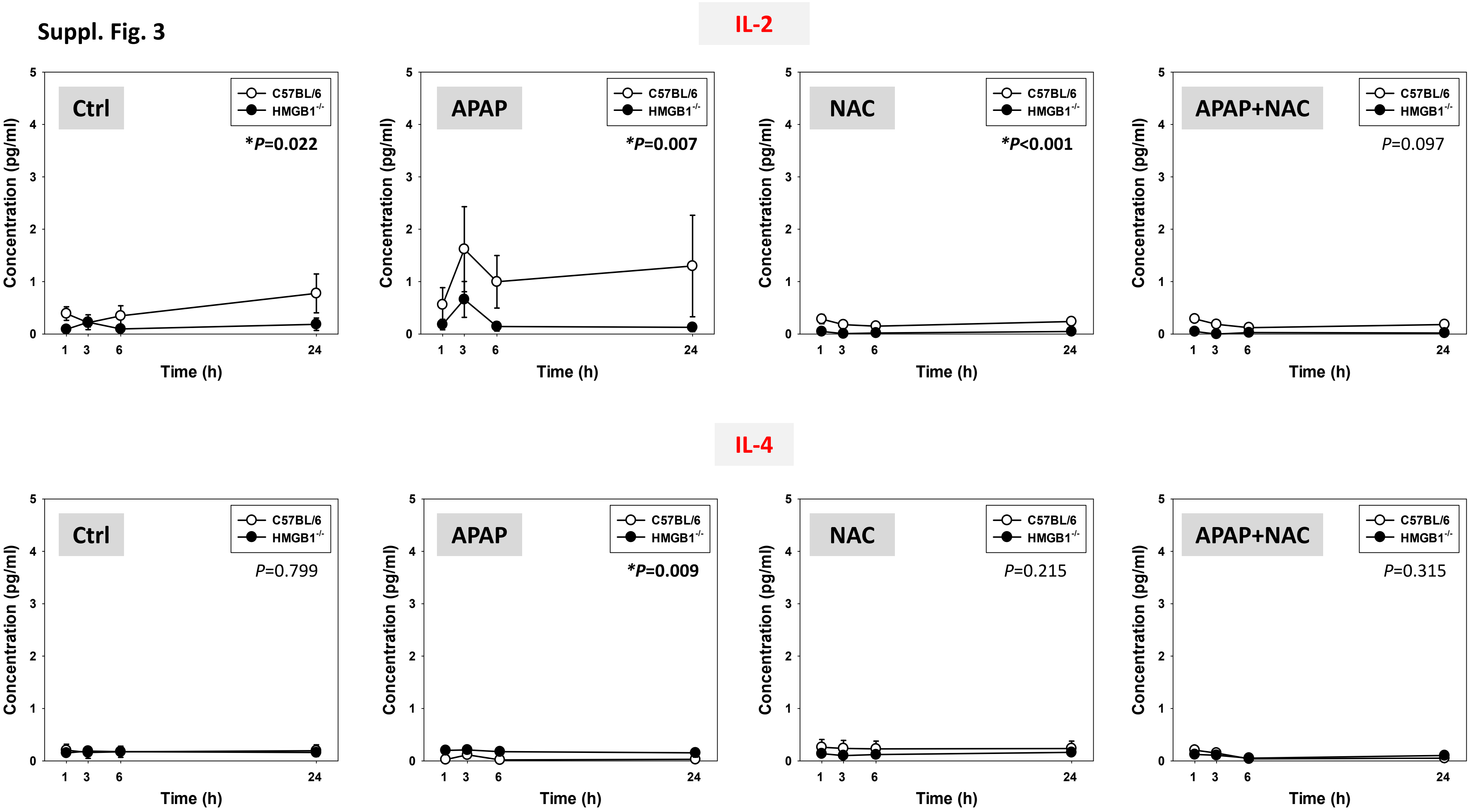
IL-2
Ctrl
C57BL/6
HMGB1-/-
*P=0.022
APAP
*P=0.007
NAC
*P<0.001
APAP+NAC
P=0.097
IL-4
Ctrl
P=0.799
APAP
*P=0.009
NAC
P=0.215
APAP+NAC
P=0.315
Concentration (pg/ml)
Time (h)
0
1
2
3
4
5
1
3
6
24

Suppl. Fig. 3

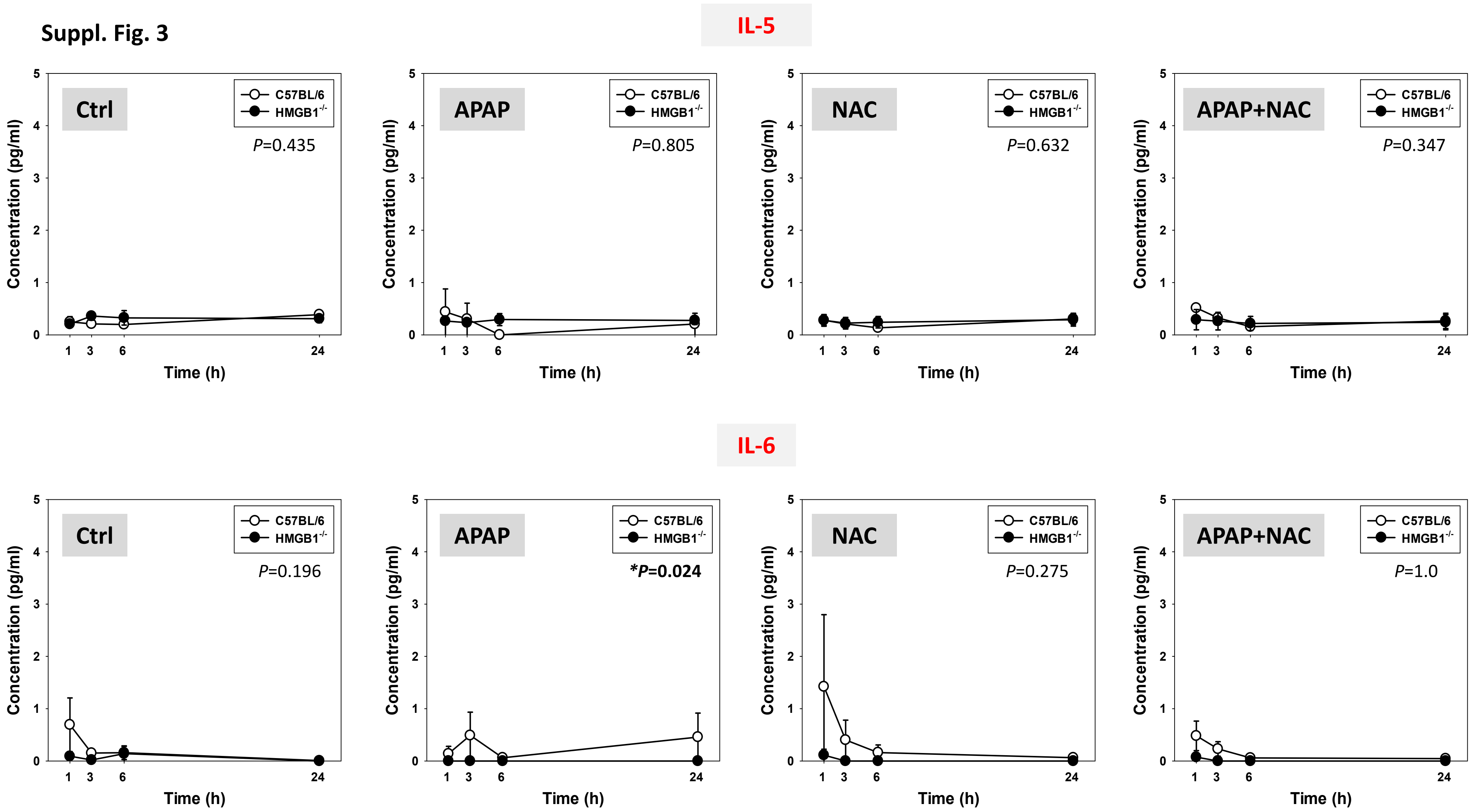
IL-5
Ctrl
APAP
NAC
APAP+NAC
C57BL/6
HMGB1-/-
P=0.435
P=0.805
P=0.632
P=0.347
IL-6
P=0.196
*P=0.024
P=0.275
P=1.0
Concentration (pg/ml)
Time (h)
0
1
2
3
4
5
1
3
6
24

Suppl. Fig. 3

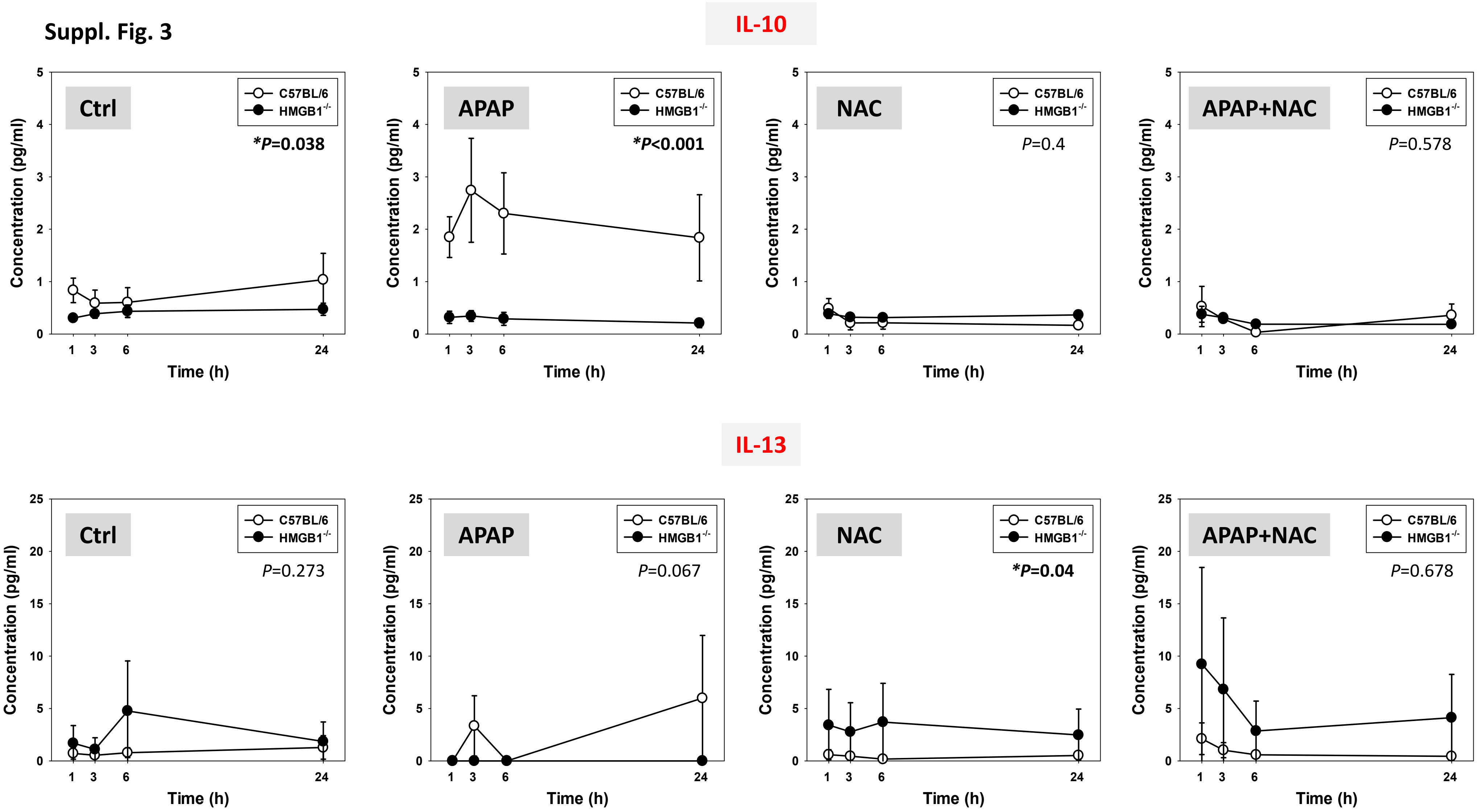

IL-10
Ctrl
APAP
NAC
APAP+NAC
C57BL/6
HMGB1-/-
*P=0.038
*P<0.001
P=0.4
P=0.578
Concentration (pg/ml)
Time (h)
IL-13
P=0.273
P=0.067
*P=0.04
P=0.678

Suppl. Fig. 3

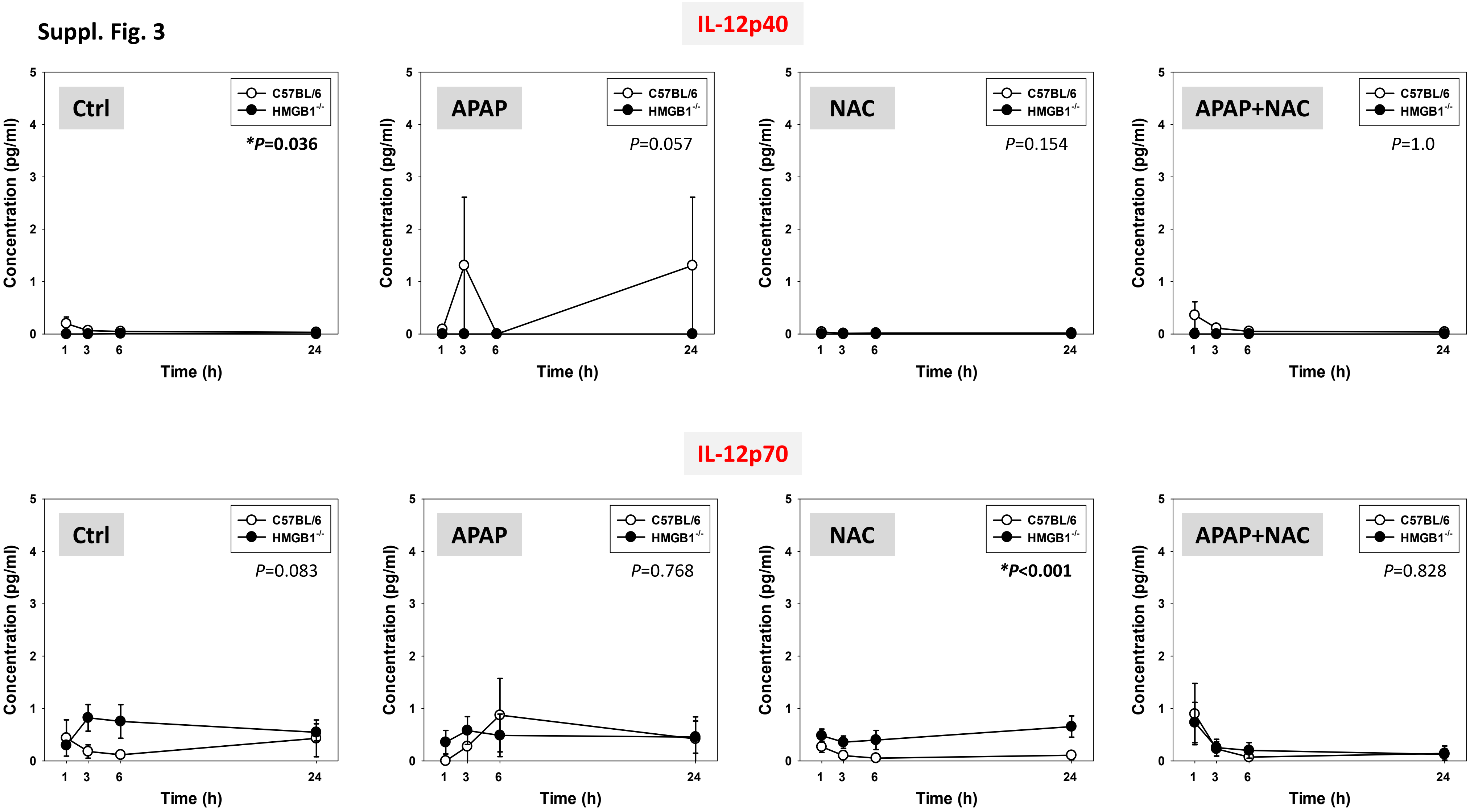
IL-12p40
Ctrl
*P=0.036
APAP
P=0.057
NAC
P=0.154
APAP+NAC
P=1.0
IL-12p70
Ctrl
P=0.083
APAP
P=0.768
NAC
*P<0.001
APAP+NAC
P=0.828
C57BL/6
HMGB1-/-
Concentration (pg/ml)
Time (h)
0
1
2
3
4
5
1
3
6
24

Suppl. Fig. 3

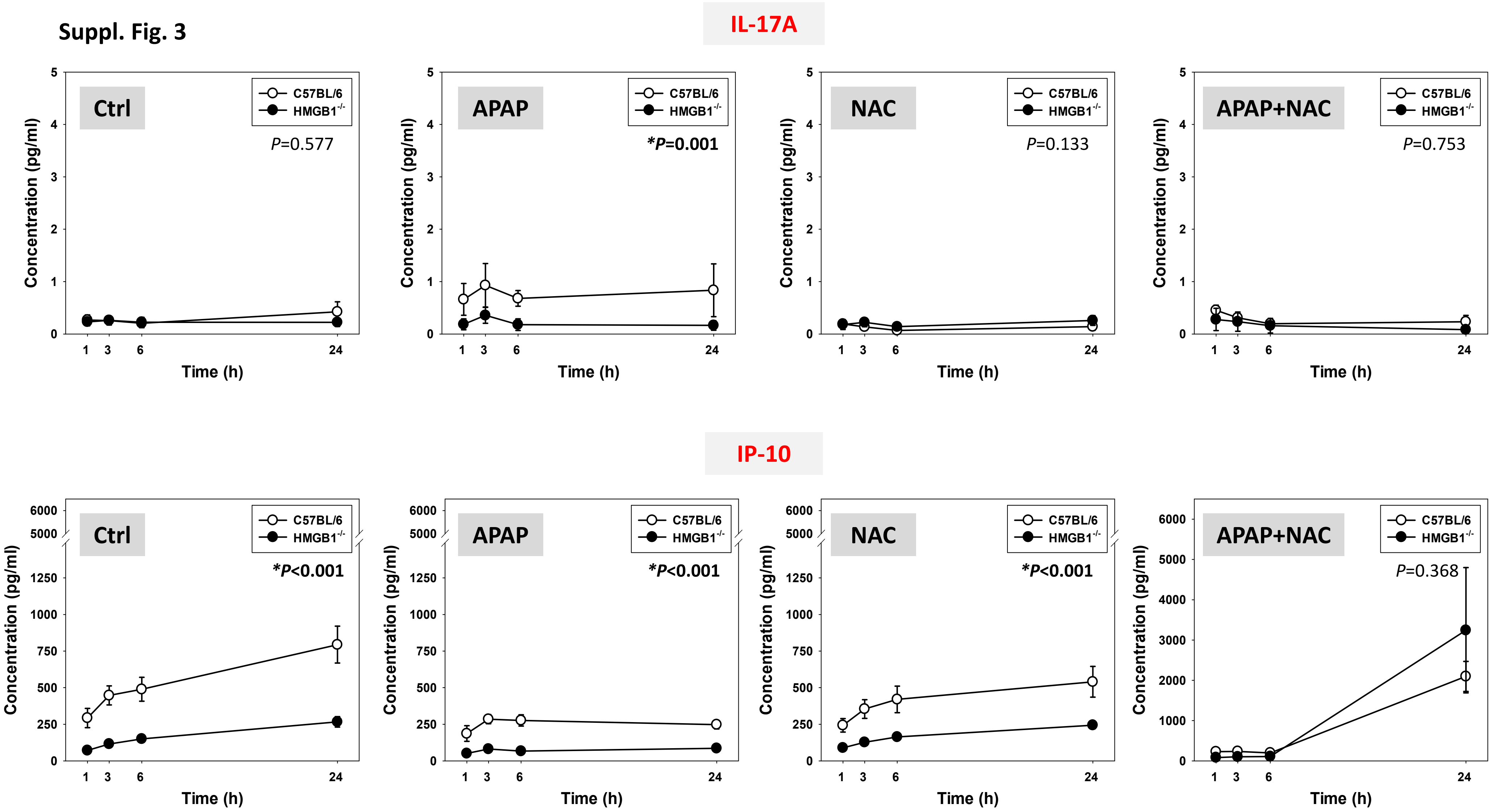
IL-17A
Ctrl
APAP
NAC
APAP+NAC
C57BL/6
HMGB1-/-
P=0.577
*P=0.001
P=0.133
P=0.753
Concentration (pg/ml)
Time (h)
IP-10
*P<0.001
*P<0.001
*P<0.001
P=0.368

Suppl. Fig. 3

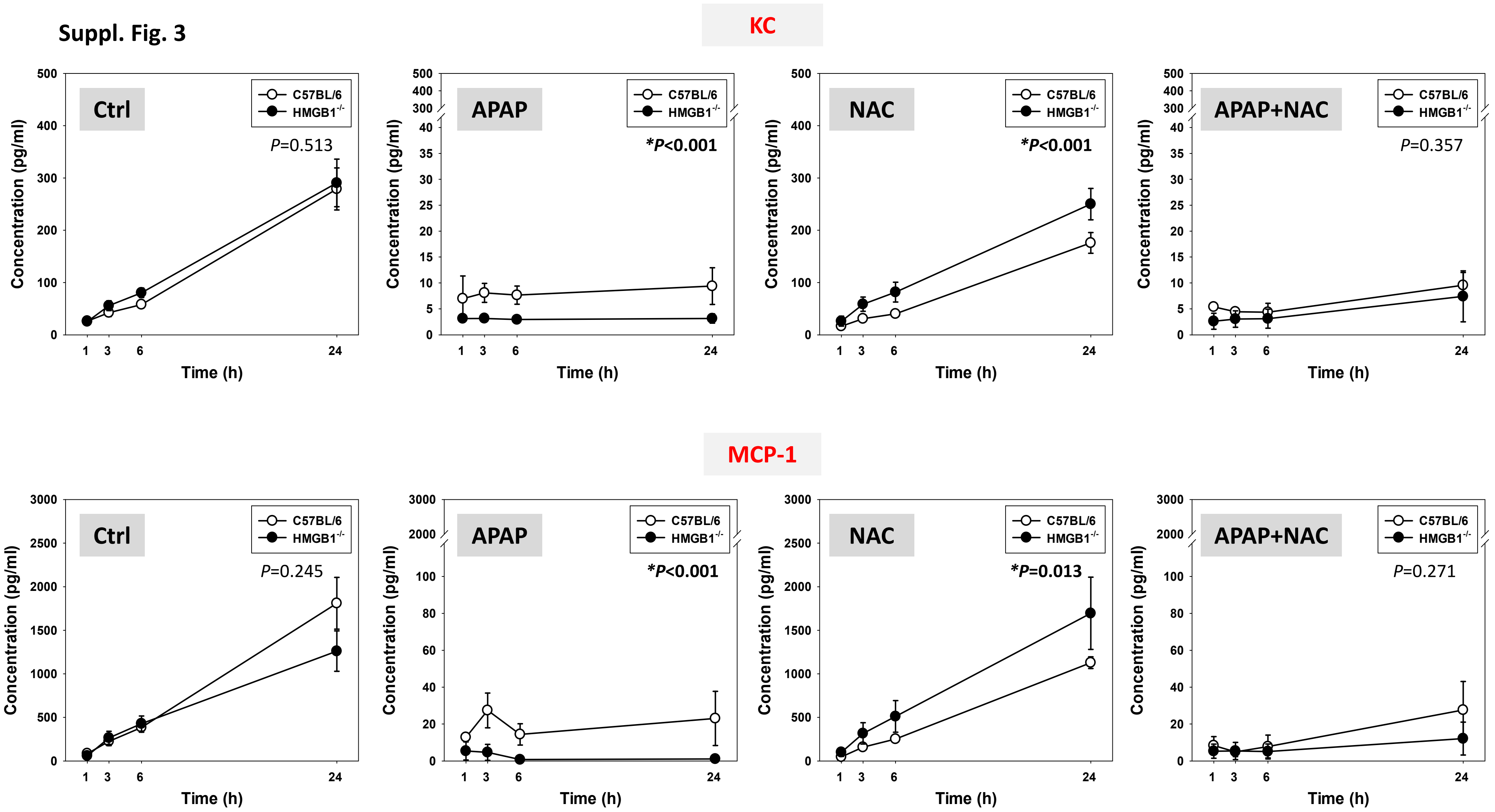
KC
Ctrl
C57BL/6
HMGB1-/-
P=0.513
APAP
*P<0.001
NAC
*P<0.001
APAP+NAC
P=0.357
MCP-1
Ctrl
P=0.245
APAP
*P<0.001
NAC
*P=0.013
APAP+NAC
P=0.271
Concentration (pg/ml)
Time (h)

Suppl. Fig. 3

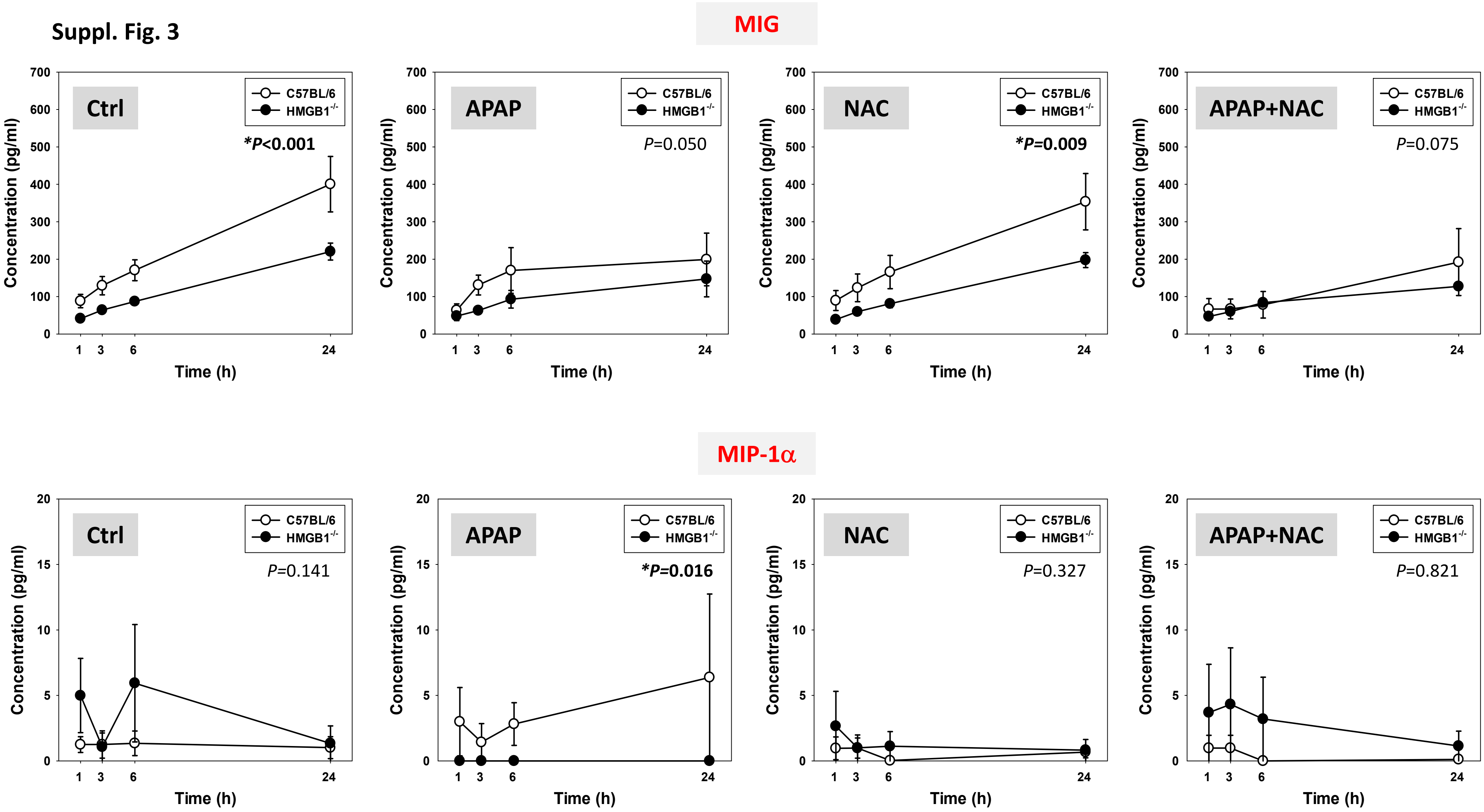
MIG
Ctrl
APAP
NAC
APAP+NAC
C57BL/6
HMGB1-/-
*P<0.001
P=0.050
*P=0.009
P=0.075
Concentration (pg/ml)
Time (h)
MIP-1α
P=0.141
*P=0.016
P=0.327
P=0.821

Suppl. Fig. 3

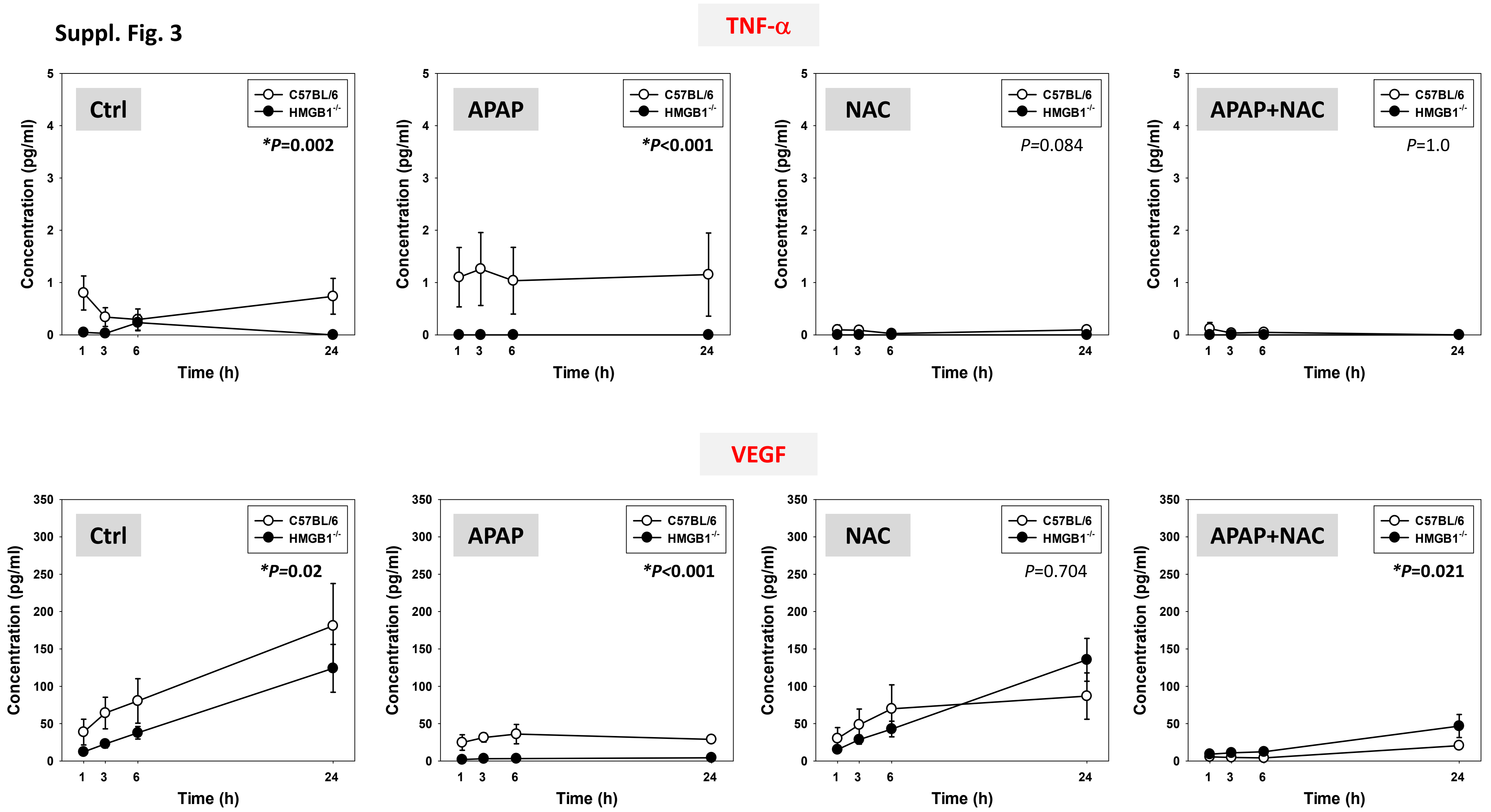
TNF-α
Ctrl
*P=0.002
APAP
*P<0.001
NAC
P=0.084
APAP+NAC
P=1.0
VEGF
Ctrl
*P=0.02
APAP
*P<0.001
NAC
P=0.704
APAP+NAC
*P=0.021
C57BL/6
HMGB1-/-
Concentration (pg/ml)
Time (h)

Suppl. Fig. 3

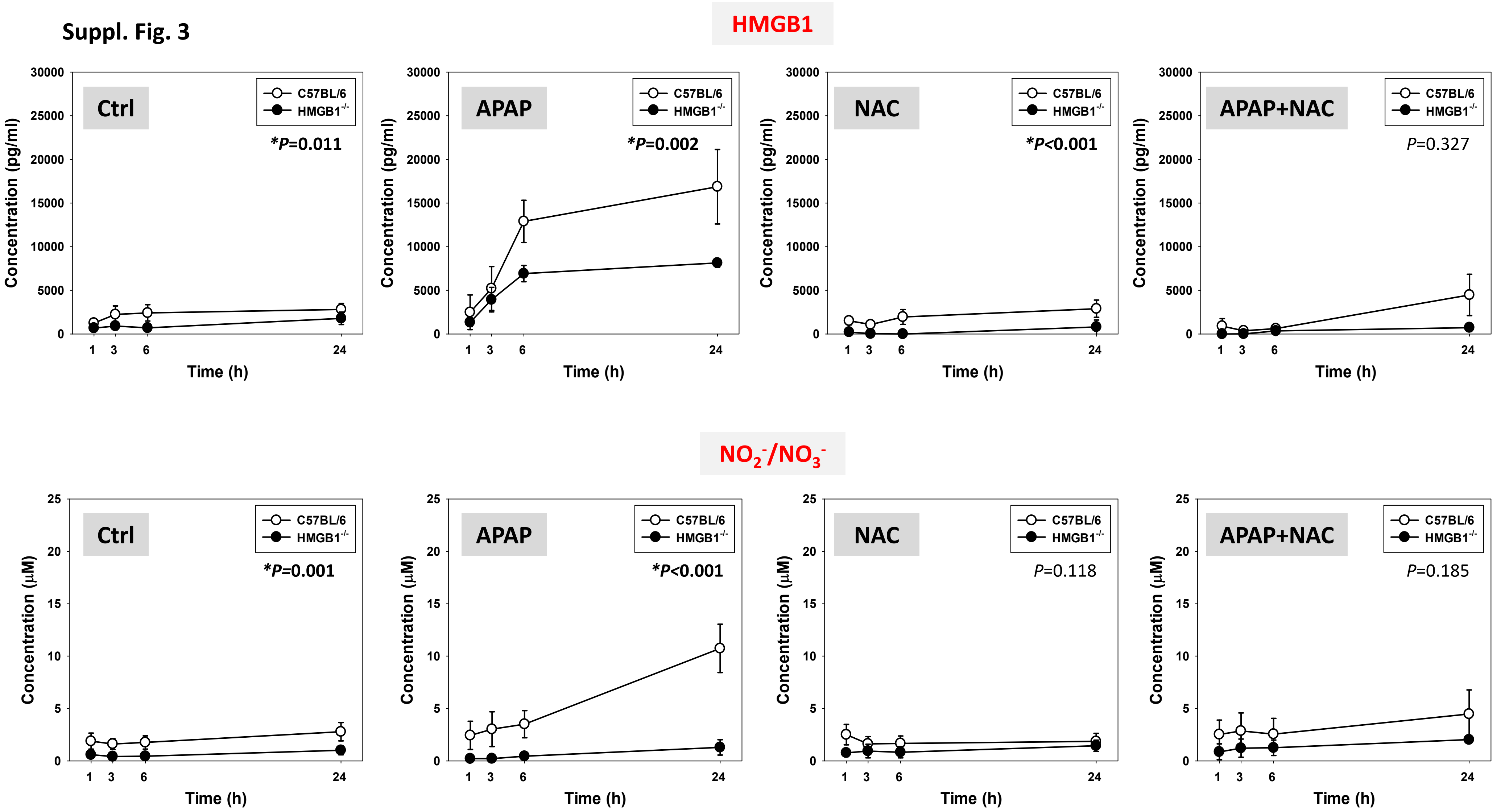

HMGB1
Ctrl
APAP
NAC
APAP+NAC
C57BL/6
HMGB1-/-
*P=0.011
*P=0.002
*P<0.001
P=0.327
Concentration (pg/ml)
Time (h)
NO2-/NO3-
*P=0.001
*P<0.001
P=0.118
P=0.185
Concentration (μM)
